# Generative models for antimicrobial peptide design: auto-encoders and beyond

**DOI:** 10.1186/s13040-026-00558-w

**Published:** 2026-05-09

**Authors:** Lukas Beierle, Julian Hahnfeld, Alexander Goesmann, Reihaneh Mostolizadeh, Franz Cemič

**Affiliations:** 1 Bioinformatics and Systems Biology, Justus-Liebig-University Giessen, Ludwigsplatz, Giessen, Hesse 35390 Germany; 2 Department of Computer Science, University of Applied Sciences Giessen, Gutfleischstrasse, Giessen, Hesse 35390 Germany; 3 Max-Planck-Institute for Terrestrial Microbiology, Marburg, Hesse 35043 Germany

**Keywords:** Antimicrobial peptides, Generative models, Deep learning, Variational auto-encoders, Wasserstein auto-encoder, Language models

## Abstract

**Background:**

As the number of multi-resistant pathogens grows rapidly, new strategies to accelerate the development of antimicrobial drugs are urgently needed. A promising candidate class of new antibiotics are antimicrobial peptides, showing a lower tendency to induce antibiotic resistance. High-throughput in silico strategies for candidate mining, such as generative deep learning algorithms, have become increasingly popular over the last few years and offer novel approaches to peptide discovery.

**Methods:**

This study presents a comparative analysis of the generative performance of deep learning models for generating novel antimicrobial peptides. The models examined include Variational Auto-Encoders, a Wasserstein Auto-Encoder, a Recurrent Neural Network, and a Language Model. The primary focus of this study is the systematic comparison and evaluation of those models and their sampling options to identify the most suitable model and sampling strategy combination for different use cases.

**Results:**

All models generated peptides with physicochemical profiles similar to natural antimicrobial sequences. Auto-encoders performed best overall, with the Wasserstein auto-encoder generating the most diverse and compositionally balanced peptides. In contrast to an unregularized baseline model, embedding-space analyses confirmed that the auto-encoders did not overfit. In addition, evaluating AMP predictors on data biased toward specific peptide properties revealed strong, model-specific preferences. Together, these results underscore the need to tailor models and evaluation metrics to each design objective.

**Conclusion:**

The present study investigates the strengths and weaknesses of various generative models for antimicrobial peptides. Practical recommendations for the combination of model type and sampling strategy are provided for individual applications.

**Supplementary Information:**

The online version contains supplementary material available at 10.1186/s13040-026-00558-w.

## Introduction

It is indisputable that there is an urgent need for the development of novel antibiotic agents and alternative treatment strategies to combat infectious diseases. Since the traditional development of antibiotics is associated with high costs in terms of time and money, new strategies have to be developed  [1]. Infections with drug-resistant pathogens remain a significant global health concern, impacting millions of lives annually. Accurately determining the case and death numbers associated with these infections presents a complex challenge due to variations in healthcare quality, reporting standards, and accessibility across different regions.

A recent study examined older forecasts of cases and deaths and incorporated historical trends and future predictions in its data. This study’s findings indicate that, from 2024 until 2050, the number of deaths attributable to AMR could exceed 39 million  [2, 3]. Among the most concerning contributors to this crisis are the so-called ESKAPE pathogens  [4–6], a group of bacteria known to have a high capacity to develop resistance to standard antibiotic therapies.

The term “ESKAPE” encompasses *Enterococcus faecium*, *Staphylococcus aureus*, *Klebsiella pneumoniae*, *Acinetobacter baumannii*, *Pseudomonas aeruginosa* and *Enterobacter species*. Often *Escherichia coli* is also considered a member of this group. Due to their multifaceted resistance mechanisms, these pathogens are prominent causes of nosocomial infections. These include (i) the production of antibiotics-degrading enzymes, (ii) the adaptation of efflux pumps that expel antibiotic substances from the bacterial cells, and (iii) modifications in membrane composition  [7–10]. Due to the high clinical relevance of this group of pathogens, the present work focuses on these target organisms.

Antimicrobial peptides (AMPs) are a particularly promising class of new antibiotics. These peptides are typically short, consisting of 10 to 50 amino acids, and are characterized by their ability to disrupt the membrane integrity of microbial cells, leading to cell death  [6, 9]. Furthermore, it is well known that bacteria are less prone to develop resistance against antimicrobial peptides (AMPs) than classical antibiotics. This is mainly due to the fact that AMPs can act on different hydrophobic and/or polyanionic targets  [11–13]. AMPs are found in almost all living organisms, from bacteria to humans, indicating their evolutionary importance as a first defense against pathogenic microorganisms  [14]. The mechanisms of action of antimicrobial peptides frequently involve targeting and permeabilizing microbial membranes  [15], yet they are also capable of interacting with intracellular targets, disrupting vital processes within pathogens  [16]. These multifaceted mechanisms of action render AMPs highly effective against a broad spectrum of microorganisms, including bacteria, fungi, viruses, and parasites. Moreover, AMPs have demonstrated efficacy against antibiotic-resistant strains, underscoring their potential as alternative therapeutic agents in an era of increasing antibiotic resistance  [9].

Generative deep learning offers significant advantages in developing novel AMPs as antibiotics. It accelerates the discovery process by rapidly generating diverse peptide sequences, which increases the likelihood of finding effective candidates  [1]. Generative deep learning helps design peptides less susceptible to resistance mechanisms by creating novel sequences.

Overall, this cost-effective and efficient approach offers a promising avenue for developing new antibiotics against drug-resistant pathogens. In recent years, there has been great progress in computational biology, particularly in the development of deep generative models. Early efforts in this area relied on Recurrent neural networks (RNNs) to capture sequential patterns and generate peptide-like sequences. Müller et al.  [17] demonstrated the ability of RNN-based models to generate functional AMPs with desired biological properties.

As generative models evolved, more complex architectures such as variational autoencoders (VAEs) and Generative adversarial networks (GANs) were introduced  [18, 19]. While GANs can generate highly realistic sequences, they are often more challenging to train due to issues such as mode collapse and instability during training  [18, 20]. In contrast, VAEs offer a more stable training framework. Advanced variants of VAEs, including WAEs, have been shown to generate peptide sequences of higher quality, as demonstrated in the research conducted by Das et al.  [1].

The majority of studies in this domain currently focus on VAEs  [21–26] or advanced variants, such as WAEs  [1, 27, 28]. In addition, the model proposed by Szymczak et al.  [29] introduces an innovative hybrid approach incorporating multi-objective optimization techniques. This technique allows more precise control of peptide properties and activity during training.

Recently, other generative approaches based on diffusion models, such as ProT-Diff  [30] and AMP-Diffusion  [31], as well as flow-matching methods  [32], have been developed, yielding promising results. The recent work of Menard et al.  [33] and Pikalyova et al.  [28] demonstrated the considerable potential of auto-encoder-based models, when employed in conjunction with advanced sampling strategies and latent space optimization.

The advent of transformer-based models, as described in the seminal research paper “Attention is All You Need” by Google DeepMind  [34], has led to novel approaches in the domain of sequence generation. These architectures leverage self-attention mechanisms to capture long-range dependencies in sequences, enabling more sophisticated peptide designs with enhanced functional properties.

In addition, there are greedy methodologies, as demonstrated by the work of Wan et al.  [35], where the authors mined the entire space of peptides of a specified length to identify new antimicrobial peptides. Furthermore, the increasing availability of diverse datasets from genomics and proteomics has resulted in the development of novel methodologies for identifying AMPs.

To illustrate this point, Santos-Júnior et al.  [36] have developed a workflow that facilitates screening genomes and metagenomes with a specific focus on AMPs. King et al.  [37] searched the human microbiome for peptide-based antimicrobials.

Additionally, Maasch et al.  [38] have successfully mined the proteomes of extinct animals to generate new antimicrobial peptides.

A comprehensive review of contemporary methodologies and the prevailing challenges in the identification of AMPs is provided in the review articles by Brizuela et al.  [39], and Szymczak et al.  [40].

In a recent study, Zhuo et al. conducted a comprehensive review of contemporary AMP databases  [41, 42].

In this study, we develop and implement various deep-learning-based methods for antimicrobial peptide generation. In addition, we propose suitable use cases for the methods developed, accompanied by pragmatic recommendations for future projects in this domain.

## Methods

### Data collection and preprocessing

#### Training data

Active AMP sequences were collected from the following publicly available databases: CAMPR4  [43], DBAASP  [44], dbamp  [45], PlantPepDB  [46], APD3  [47], and LAMP  [48]. It should be noted, however, that the sequences from the LAMP database were not directly obtained from the database but from the work of Bournez et al.  [49], as the LAMP web service was no longer available. The date of the last database state is provided in Supplementary Table 2.

The collected sequences were filtered according to the following criteria: They were required to contain only canonical amino acids. The sequence length was required to be between 3 and 36, enabling uncomplicated solid-state peptide synthesis. Furthermore, their minimum inhibitory concentration (MIC) or inhibitory concentration (IC) was required to be $\leq {35}\,{\mu}\textrm{M}$. Sequences exhibiting MIC values that differ by more than a factor of three for the same target organism were excluded from further analysis. Sequences with MIC values for different target organisms were retained for cases where the median MIC across all targets was $\leq 35\,{\mu}\textrm{M}$.

In the case of the auto-encoder-based models, the training dataset was randomly split by 90:10. The second part is used to assess the reconstruction capabilities of the auto-encoders. This dataset is henceforth designated as the reconstruction dataset.

The RNN and the language model were trained on the entire dataset. The rationale for this decision is that RNNs and Language models (LMs) cannot be used for template-based sequence reconstruction, like auto-encoders. However, the reconstruction dataset might contain homologous sequences, which will artificially inflate reconstruction accuracy values and should therefore be regarded only as proxies. The reconstruction accuracy is not included in the qualitative performance measures of the auto-encoders used in this work.

#### Comparison data

Four datasets were created for comparison of the generated sequences. The first dataset contains sequences from the UniProt  [50] database, which are assumed to be non-AMPs. The advanced search feature of the UniProt web interface was utilized to exclude potential AMPs from the selection. The following keywords were used to exclude peptides: Antimicrobial (0929), Fungicide (0295), Antibiotic (0044), Tumor Suppressor (0043), and Antiviral Protein (0930). Additional restrictions were applied to search fields “pharmaceutical or biotechnological use,” and the protein existence level was utilized to exclude peptides with uncertain evidence. The range of sequence length was constrained from 3 to 36. The UniProt dataset encompasses 6.385 sequences.

The second dataset comprises random sequences of equal length and amino acid distributions similar to those in the training dataset. Care was taken to ensure that the second dataset and the training dataset were of equal size.

The third dataset was created by random sampling 40.000 sequences of lengths 3–36 from an amino acid distribution with equal probabilities for all amino acids. Duplicate samples were removed.

The fourth dataset consists of artificially generated amphipathic helices, characterized by a high hydrophobic moment. This approach was adopted from Müller et al.  [17].

The datasets referenced herein are primarily used to compare the prediction outcomes outlined in Sect. 3.5.

### Sequence encoding

The OneHot encoding scheme was used in all auto-encoder-based models. Prior to this, all sequences were adjusted to a uniform length of 36. Hyphen characters of appropriate length are used as prefixes to extend smaller sequences.

According to the model’s documentation on KerasHub  [51], a specific padding character was employed in the context of language models. This character was denoted by the token ”[PAD]”. In addition to this, the token ”[BOS]” was inserted at the beginning of each sequence to demarcate its starting character. For this purpose, the StartEndPacker from the KerasHub library was used. In contrast to the other models, the language model utilized a distinct encoding method. The TokenAndPositionEmbedding layer from KerasHub was used for a positional embedding. The training sequences utilized by the RNN were processed following the methodology outlined by Müller et al.  [17].

### Evaluation metrics

In addition to employing a subset of peptide descriptors associated with AMP activity, Supplementary metrics were utilized to quantify the novelty, diversity, and authenticity of template-free-generated sequences. The metrics were calculated using the libraries peptides.py  [52] and seqme  [53]. The diversity metric is defined as the normalized pairwise Levenshtein distance between the top k sequences; in this work, $k=10$.

Novelty is defined as the fraction of sequences not occurring in a set of references. Uniqueness is the fraction of unique sequences. The authenticity of a sequence is determined based on embeddings, defined as the ratio of sequences whose nearest neighbor in the training set is more similar to another training sample than to the sequence itself. Similarity between the distributions of peptide properties is quantified by the maximum mean discrepancy (MMD). Embeddings based on the NEAR  [54] model were computed using the mininear Python library  [55]. The aforementioned metrics are displayed in Table 1.

### Generative models

#### Variational auto-encoders

VAEs, first described by Kingma et al.  [56], are an extension of classical auto-encoders based on variational inference. These models consist of an encoder $q_\phi$ and a decoder $p_\theta$, each represented by a neural network with parameter sets denoted by $\phi, \theta$, respectively. In a variational inference setting, the objective is to approximate the unknown distribution $p(x)$ of the given dataset. The VAE achieves this by approximating $p(x)$ with the marginal likelihood $p_{\theta}(x)$.

For this purpose, the encoder maps each data point $x$ into a continuous latent space $p(z)$, which is forced to be standard normally distributed. During training, the VAE aims to maximize the evidence lower bound (ELBO) of the marginal likelihood $p_\theta(x)$, which is equivalent to minimizing the Kullback-Leibler divergence (KL) between the posterior approximated by the decoder $q_\phi(x \mid z)$ and the true posterior $p_\theta (z \mid x)$.

1 $$\mathcal{L}_{\mathrm{VAE}}(\theta, \phi) = \mathbb{E}_{q_{\phi(z \mid x)}}[log \; p_{\theta}(x \mid z)] - D_{KL}(q_{\phi}(z \mid x) \mid \mid p_{\theta}(z))$$

#### Wasserstein auto-encoder

WAEs are an extension of VAEs proposed by Tolstikhin et al.  [20]. Instead of using variational inference as an objective, WAEs rely on optimal transport $W_c(P_X, P_G)$ where $P_X$ is the actual data distribution and $P_G$ is the approximation by the model. $G(Z)$ denotes a sample from the decoder. The distance between the input and the reconstructed data is measured by a cost function $c(P_X, P_G)$ chosen as the mean squared error in this work.

Instead of computing a pointwise approximation to the latent prior $P_Z$ as is usually done by VAEs, the WAE forces a continuous mixture $Q_Z :- \int Q(Z \mid X) dP_X$ to match $P_Z$  [20]. Rather than using KL, the WAE is regularized with a MMD term $D_Z(Q_Z, P_Z)$ in Eq. 2  [20, 57]. Specifically, as proposed by Tolstikhin et al.  [20], we used an inverse-quadratic mean kernel to compute the MMD and introduced $\lambda$ as a scaling parameter. This leads to the optimization problem given by Eq. 2.

2 $${{\mathrm{D}}_{{\mathrm{WAE}}}}({P_X},{P_G}): = \mathop {\inf }\limits_{Q(Z\mid X) \in \mathcal{Q}} {\mathbb{E}_{{P_X}}}{\mathbb{E}_{Q(Z\mid X)}}[c(X,G(Z)] + \lambda \cdot {D_Z}({Q_Z},{P_Z})$$

#### Recurrent neural network

As described by Müller et al.  [17], RNNs were initially used in natural language processing and can capture medium-range dependencies in sequences. Later on, more advanced variants such as Long-Short-Term-Memory (LSTM) cells  [58] and Gated-Recurrent-Units (GRUs)  [59] were developed to overcome certain limitations of RNN, such as vanishing or exploding gradients  [17, 58].

GRU units were developed to simplify LSTM cells. RNNs can also be used to generate new sequences. For this, one or more starting characters can be provided to the model. Then, it recursively predicts the most probable characters based on the representation learned during training. As an objective function, the RNN uses categorical cross-entropy.

#### Language model

Language models (LMs) are a type of architecture based on the seminal paper “Attention is all you need” by Google DeepMind  [34], which introduced the transformer architecture for natural language modeling. Various implementations designed for protein sequence generation were published  [60–62]. Inspired by the architecture of ProtGPT-2  [62], a transformer-based decoder-only model, a small language model for AMP sequence generation was also developed in this study.

Language models typically undergo extensive training on very large datasets, often with prolonged pre-training periods requiring substantial resources. In contrast, the present study uses a smaller dataset, leading to the decision to implement a compact language model. This model has a single TransformerDecoder layer with two attention heads, an embedding dimension of 32, an intermediate dimension of 32, and a dropout rate of 0.1. The LM’s sampling procedures differ from those of the RNN, VAE, and WAE. The LM uses its own sampling algorithms, provided by the KerasHub library.

### Model architectures and implementations

The models introduced in Sect. 2.4 (a WAE, an RNN, and four VAEs with different regularization schemes) were implemented alongside a small LM.

**Fig. 1:**
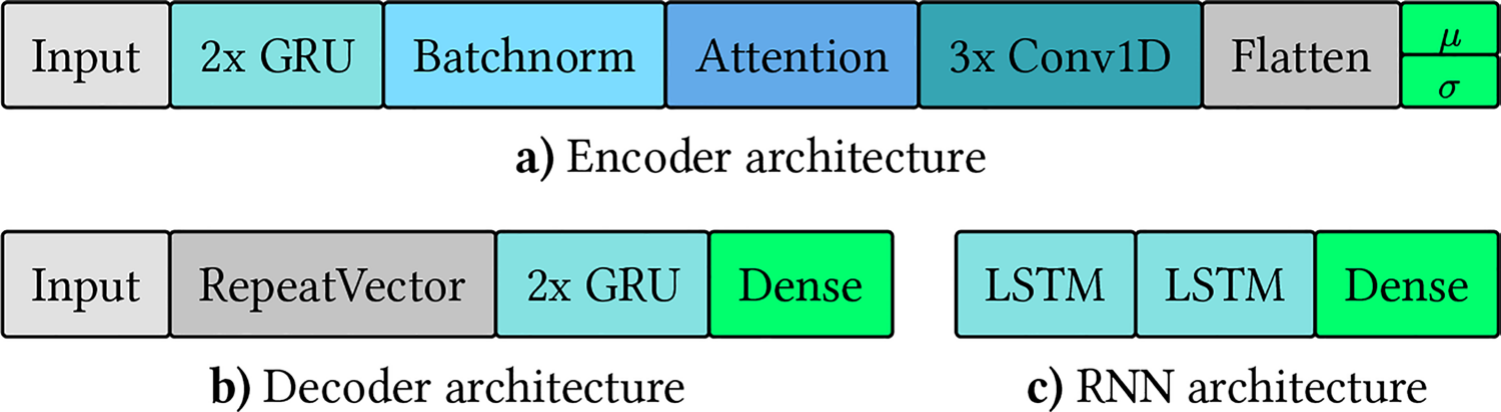
Visualization of the architecture of RNN, encoder and decoder. The architectures used for the encoder and decoder of the VAEs, WAE, and the RNN (left to right). (**a**) The encoder has an input layer, two GRU layers, and a batch normalization layer. An attention layer performs self-attention. Three 1D convolution layers are used, followed by a flatten layer, with additional dense layers representing the latent distribution. The WAE uses only one dense layer instead of two for the latent distribution. (**b**) The decoder’s architecture comprises an input layer, a RepeatVector layer to match the required input shape of the two consecutive GRU layers, and a dense layer. (**c**) The RNN consists of two interconnected LSTM layers and a dense layer that functions as the output layer

Figure 1 shows a visualization of the architecture of the RNN, as well as the encoder and decoder used by the WAE and VAE. The layer names correspond to the Keras version 2 documentation. Layers of the same type are color-coded. The RNN consists of two interconnected LSTM layers, with 256 units each. These layers are followed by a dense layer comprising 21 units and a softmax activation function. The encoder architecture presented here is used by the WAE and VAE, with a minor difference explained below. The encoder is composed of an input layer, two GRU layers with 128 units each, a batch normalization layer, an attention layer that performs self-attention, three 1D convolution layers, followed by a Flatten layer to transform the output dimension to 1D. Next two dense layers representing $\mu$ and $\sigma$ for the latent distribution are used. The WAE uses a single dense layer for the latent distribution rather than two.

The decoder utilized by VAE and WAE consists of an input layer followed by a RepeatVector layer. The functionality of this layer is to ensure that the appropriate input dimension is provided to the subsequent two GRU layers, each comprising 128 units. A dense layer with 21 units, followed by a softmax activation function, serves as the final output layer.

A summary of the used hyperparameters: batch size, number of epochs, learning rate, and the size of the latent dimension can be found in Supplementary Tab. 3 and Supplementary Fig. 11.

It is important to note that, during training, the learning rate of the WAE was reduced with an ExponentialDecay schedule from the Keras library  [63]. This approach was also employed in the original work by Tolstikhin et al.  [20].

#### Annealing schedules

A well-known problem encountered during VAE training is posterior collapse  [64]. This occurs when both terms on the right-hand side of the Eq. 1 differ in magnitudes. When the reconstruction term is assigned a greater weight than the regularization term, the decoder tends to neglect the latent variables and reconstructs the input directly. Consequently, the KL divergence then approaches zero, thereby enabling the maximization of the ELBO. However, models than fail to learn meaningful data representations. As a result, the generated data exhibits high similarity to the input data and lacks diversity.

To avoid posterior collapse in VAE models, KL annealing was used. Specifically, a scaling factor $\beta$ is introduced to the KL term of the objective function in Eq. 1. The parameter $\beta$ was iteratively modified during training as detailed by Eqs. 3, 4 and 5. In addition, a fixed $\beta = 1$ (Eq. 6) was included for comparison to a non-regularized VAE. The KL-regularization during training forces a VAE to assign greater weight to the reconstruction term from Eq. 1.

3 $$\beta_{\mathrm{cyclic}} = \begin{cases}f(\tau), & \tau \leq R \\1, & \tau > R\end{cases} \quad \mathrm{with} \quad \tau = \frac{\mathrm{mod}(t - 1, \lceil T / M \rceil) }{T / M } $$

4 $$\beta_{\mathrm{logistic}} = \frac{1 }{1 + e^{-s \cdot (epoch_{i} - epoch_{\max})}} $$

5 $$\beta_{\mathrm{linear}} = \frac{epoch_{i} }{epoch_{\max} } $$

6 $$\beta_{\mathrm{normal}} = 1$$

In Eq. 3, $t$ denotes the current training iteration number, a.k.a. epoch, $T$ is the number of epochs, $f$ is a monotonously increasing function, while $M$ is the number of annealing cycles during training, and $R$ is the proportion that is used to increase $\beta$ within one cycle. $M$ and $R$ were set to the default values as in  [65] ($M = 4, R = 0.5$). In Eq. 4, the parameter $s$ controls the steepness of the logistic curve, while $epoch_{max}$ sets its infection point and $epoch_i$ denotes the current training iteration. The parameter $s$ was set to 0.01, while $epoch_{max}$ was equal to 350.

#### Baseline model

To the best of our knowledge, the RNN from the work of Müller et al.  [17] was the first model of this type used to generate antimicrobial peptides. The model and training data were made publicly available, enabling full reproducibility. Therefore, the RNN has been selected as the baseline model for this investigation. Later, the VAE models are referenced by their annealing schedules, as shown in the following example: Cyclic (VAE-CYC), Linear (VAE-LIN), Logistic (VAE-LOG), and without annealing (VAE-N). Information about the hardware used to train the models is available in the Supplementary material.

### Sampling strategies

Different methods were used to generate putative new AMP sequences depending on the respective model.

#### Template-free sequence sampling

All models can generate peptide sequences without reliance on templates. In auto-encoder-based models, this refers to drawing a random sample of dimension $sequences \times length$ from the latent prior. The decoder maps the output to the space of peptide representations and translates it to a sequence of amino acids.

Template-free sampling by the RNN is initiated by feeding in a random start amino acid, to which further ones are added recursively until a padding character is emitted or the maximum sequence length of 36 is reached. For all models, the number of sequences generated by template-free sampling was fixed at 40.000.

#### Template-based sequence reconstruction

Template-based sequence reconstruction is a feature unique to the auto-encoder-based models used in this work. Sequences from the reconstruction dataset served as templates for the reconstruction. After training of the VAEs and the WAEs, these sequences are first OneHot encoded and then mapped to the latent space by the encoder. These representations are then fed into the decoder and translated into amino acid sequences. Using that scheme, sequences similar to the templates in terms of amino acid composition and peptide properties are generated, as will be discussed in Sect. 3.

#### Template-based multi sequence sampling

**Fig. 2:**
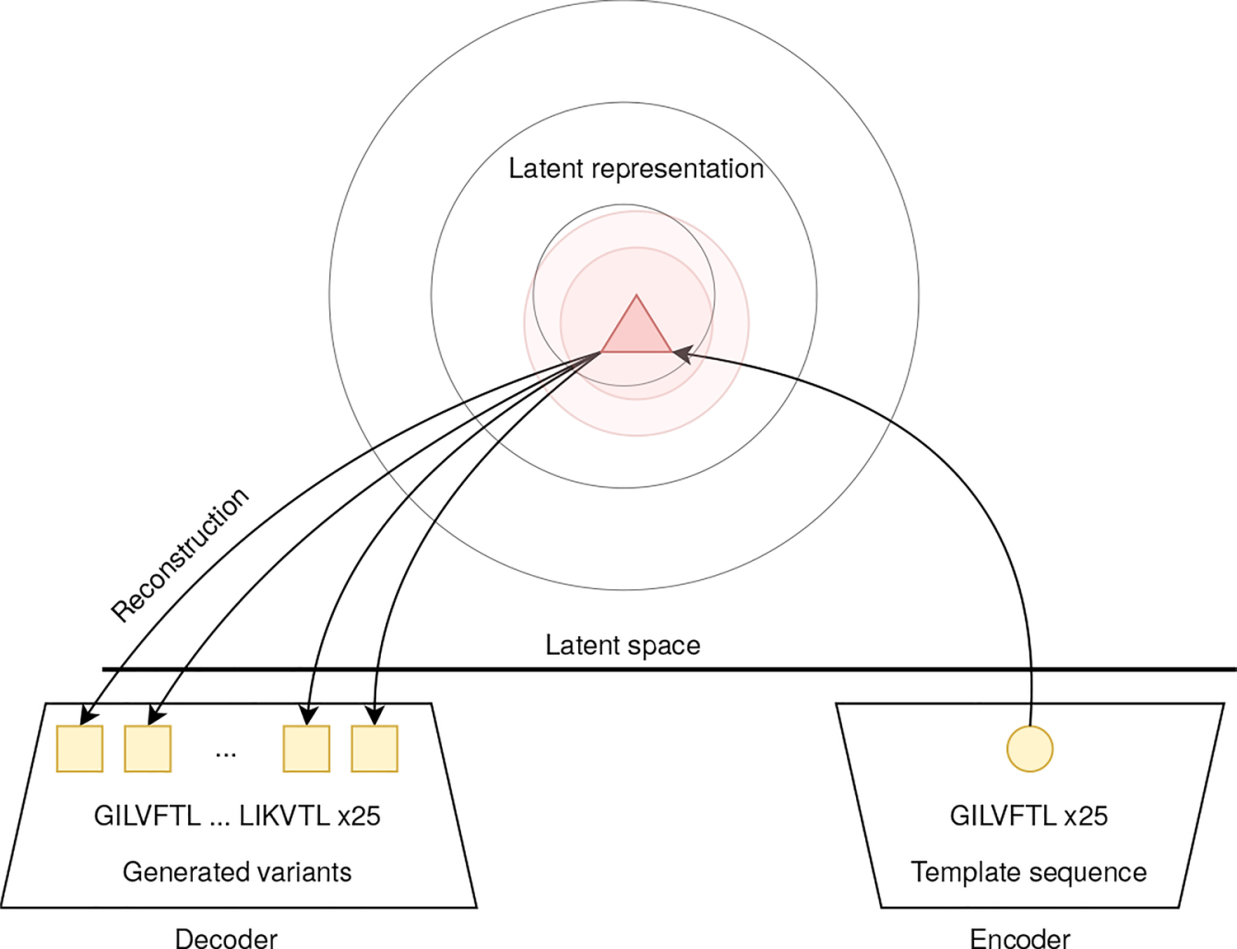
Visual representation of template-based multi sequence sampling. The exemplary input sequence GILVFTL was encoded and repeated 25 times, then mapped into the latent space by the encoder. Each input sequence is mapped to different Gaussian distributions on the latent space, where each distribution is a point-wise approximation to the ELBO. After this, the decoder reconstructs 25 individual samples from the latent distribution, resulting in different generated sequences called variants

As mentioned, VAEs approximate the latent prior $p(z)$ point-wise. Consequently, if the local approximation is sufficiently precise, multiple samples of a single sequence can be generated by repeating the reconstruction procedure, as visualized in Fig. 2. This sampling strategy is particularly interesting if the template sequence is considered a lead sequence, such as one with desired properties for which different analogs should be generated.

The strategy is contingent upon the assumption that the local approximation of the ELBO of the targeted peptide is sufficiently rich. In the absence of this condition, there will be minimal difference in the resulting samples; alternatively, the samples will be identical. For each sequence in the reconstruction dataset, a batch of 25 replicates was mapped into the latent space by the encoder. As a result, all sequences were mapped to a local distribution as indicated schematically by the red circlein Fig. 2. In the next step, a batch of equal size was sampled from the latent representation, yielding a reconstruction of the template sequence.

#### Language model samplers

The LM utilizes three distinct sampling algorithms to generate sequences. These algorithms append the next amino acid with the highest probability, as calculated by the sampler, to the sequence starting from the start token, and generate new sequences continuously. This process is then repeated. Three samplers from the KerasHub library were used. The TopPSampler with $p=0.9$, TopKSampler with $k=10$ and a temperature of 1.2. Parameter optimization was performed using grid search. For the RandomSampler, standard parameters were used.

### Sequence selection and evaluation

Generated sequences were filtered according to the following criteria: a sequence length between 3 and 36, the absence of padding characters, and no recurrence of individual amino acids more than seven times. For all valid sequences, training, and comparison datasets, the descriptors of hydrophobicity (Eisenberg scale), hydrophobic moment (Eisenberg scale), charge at pH 7, the isoelectronic point, and length were calculated using the *peptides.py*  [52] library in Python.

**Fig. 3:**
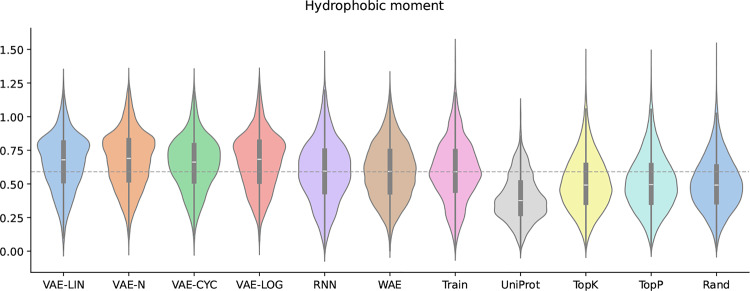
Distributions of the hydrophobic moment. Distributions of the hydrophobic moment are shown as violin plots, including median and interquartile distances. The distributions of all template-free-generated sequences, as well as the training and comparison datasets, are shown. The gray dashed line marks the median of the training datasets’ distribution for easier visual comparison

These descriptors were selected based on their correspondence to the activity of AMPs. Furthermore, they have been utilized in previous studies  [1, 17]. As illustrated in Fig. 3, the distribution of the hydrophobic moment is presented as it is considered the most important descriptor. The figures for the distributions of the remaining data are in the Supplementary section. At this moment, drawing parallels with other studies concerning the peptide characteristics of generated sequences was not feasible. This was due to an inconsistent data foundation, the non-disclosure of models and data, and a lack of reproducibility.

To facilitate comparison of auto-encoder-based models’ ability to reconstruct sequences, reconstruction accuracy per peptide position was calculated according to the method described by Renaud et al.  [27]. The reconstructed sequences were not filtered, as otherwise it would no longer be possible to assess the reconstruction’s accuracy.

### AMP activity prediction

To facilitate further identification of possible active AMPs in the generated datasets, four publicly available tools for AMP prediction were used. The neural network-based *Antimicrobial Peptide Scanner v2* from the work of Veltri et al.  [66], the recently published tool AntiBP3 by Bajiya et al.  [67], Macrel from Santos-Júnior et al.  [36] and AMPlify from the work of Li et al.  [68].

Prediction results for each tool and majority-voting are provided for template-free-generated sequences, as well as for the training data and the comparison datasets described in Sect. 2.1 The prediction results are shown in Tab. 4; more information is provided in Supplementary Tab. 8. The tools mentioned above were chosen for AMP activity prediction because all the data required to reproduce and test the model are publicly available.

### Visualization of peptide features via manifold learning

Peptide descriptors relevant to AMPs and general physicochemical properties were computed for both model-generated and known AMPs from the training set (Supplementary Tab. 5). Dimensionality reduction and visualization were conducted to reveal global and local distribution patterns, enabling qualitative assessment of how generated sequences align with or differ from the training manifold. Visualization of the reduced feature space also enables investigation of potential model overfitting.

Two complementary nonlinear dimensionality reduction techniques were employed: Uniform manifold approximation and projection (UMAP)  [69] and t-distributed Stochastic Neighbor Embedding (t-SNE)  [70]. The *umap-learn* implementation  [71] was used for UMAP, and the *opentsne* library  [72] was used for t-SNE. The resulting two-dimensional embeddings were visualized with the Bokeh library  [73]. To balance local and global structure in the reduced space, specific parameters for UMAP and t-SNE were adjusted. The exact parameter values are provided in Supplementary Tab. 6.

**Fig. 4:**
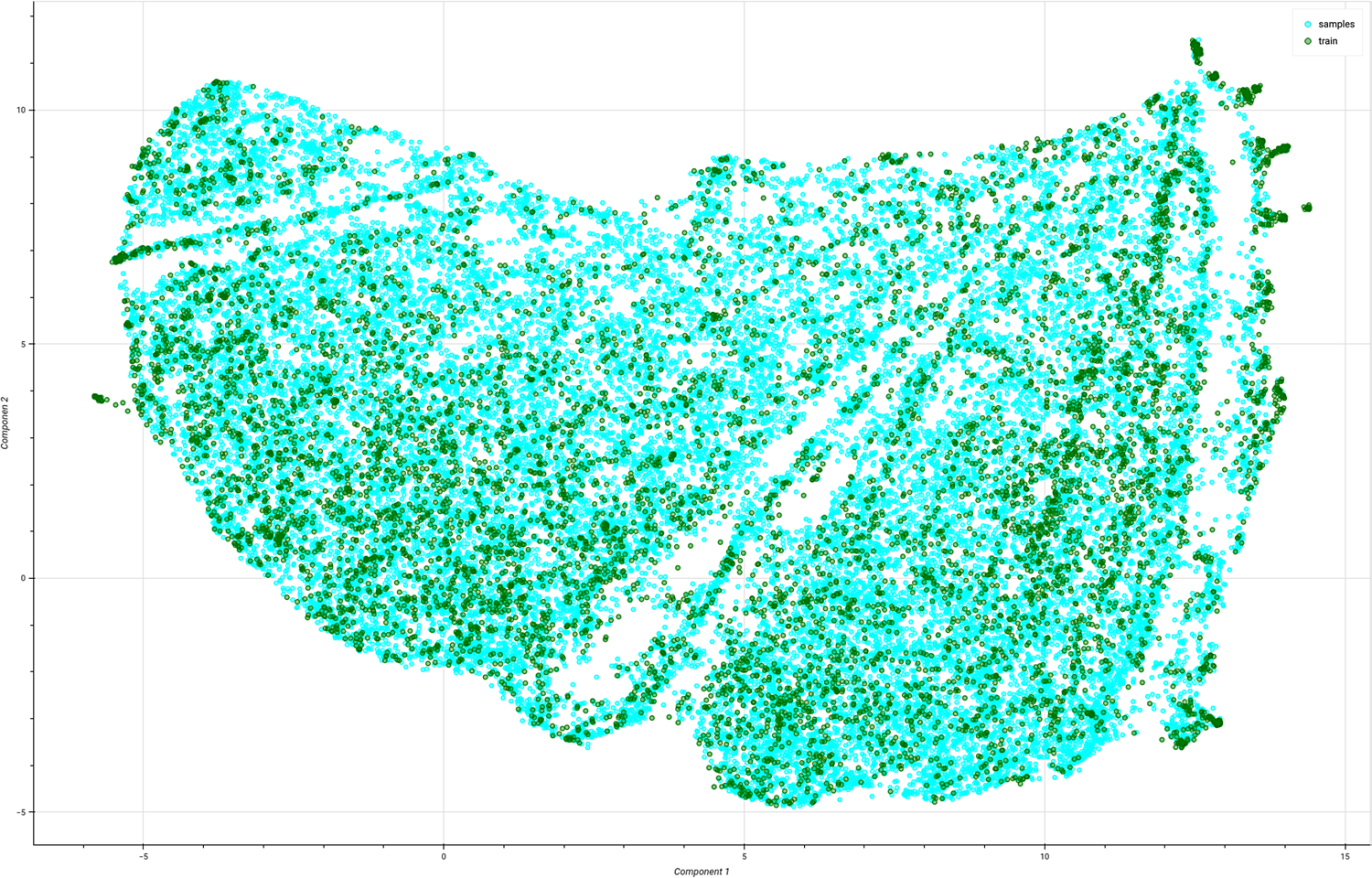
Visualization of the peptide property clustering performed by UMAP two-dimensional UMAP embedding of feature vectors representing multiple physicochemical properties of generated AMPs. Cyan points correspond to sequences generated by the Wasserstein Autoencoder post-filtering, while green points denote sequences from the training dataset. The overlapping distributions reflect the WAE’s ability to produce novel peptides that span the chemical diversity of known AMPs. Local density variations indicate regions of shared functional or structural characteristics, while the absence of isolated clusters suggests effective coverage and generalization of the model. This visualization supports that generated peptides inhabit a similar biochemical manifold to the training data, underscoring the model’s potential for guiding de novo AMP design

Figure 4 presents a UMAP visualization of WAE-generated peptides, while a t-SNE visualization of the same sequences is provided in Supplementary Fig. 9. Additional t-SNE visualizations for the VAE-LIN model and the RNN are presented in Supplementary Figures. 12 and 13. Supplementary Tab. 5 contains the complete list of calculated peptide properties. To minimize length-related biases, the molecular weight descriptor was normalized by peptide length. All properties were computed using the peptides.py library  [52].

## Results

### Template-free sequence generation

**Fig. 5:**
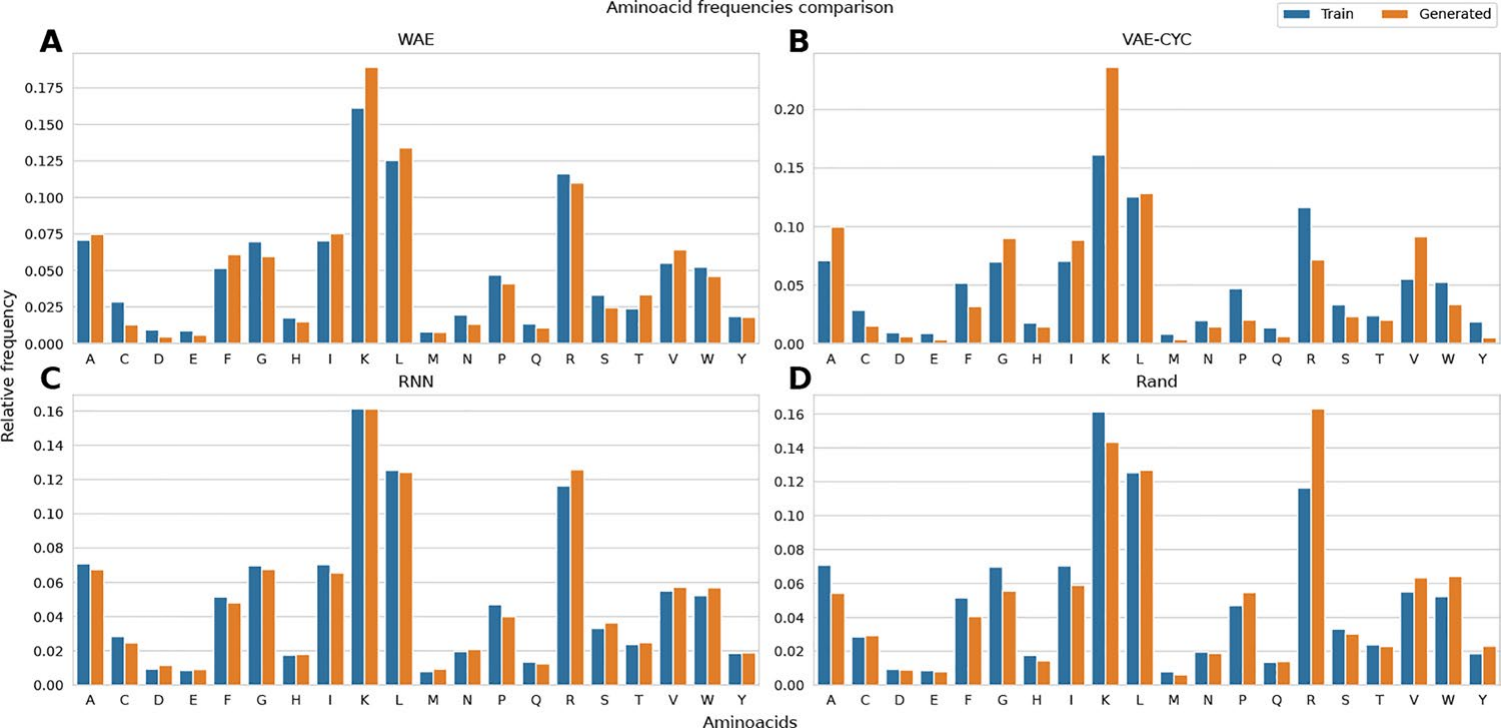
Amino acids comparison between the training set and generated sequences. Each sub-figure shows the relative frequency of individual amino acids within the training sets (blue) and the generated sequences (orange). The results for the WAE (**A**), VAE with cyclic annealing (**B**), RNN (**C**), and RandomSampler (**D**) are presented from the upper left to the lower right

In this section, template-free generated sequences are analyzed regarding amino acid composition and peptide properties associated with AMP activity. Figure 5 shows the amino acid composition for the WAE, RNN, VAE-CYC, and the LM with the RandomSampler.

A summary of mean, maximum, and minimum values, and the most prominently deviating amino acid, is shown in Supplementary Tab. 4. Regarding variations in amino acid composition, the WAE demonstrates the least deviation among the auto-encoder-based models. Compared to the other auto-encoders, this model shows reduced disparities in the amino acid composition for less abundant amino acids. Among the VAE-based models, the VAE-CYC features the least deviations, except for the mean deviation. In this case, the VAE-LOG model exhibits a smaller value but has higher maximum and minimum deviations.

The RandomSampler, compared with the other two samplers, shows a remarkably lower degree of deviation in amino acid composition. The RNN, on the other hand, shows an almost identical amino acid composition for generated and training sequences. This is due to the modest size of the training dataset, and the absence of regularization in this model. Supplementary Figures 4 and 5 present the plots for the additional models. Supplementary Table 7 provides the Wasserstein distances between the amino acid distributions depicted in Fig. 5.

Figure 3 shows the hydrophobic moment distributions for the generated sequences, the training set, and the UniProt comparison dataset, using violin plots. The median of the training set is shown as a gray dashed line for comparison. As demonstrated in Fig. 3, an upward shift of the mode positions of the hydrophobic moment distributions is evident for the VAE-LOG, VAE-N, and VAE-LIN models.

This phenomenon is partially attributable to the over-representation of the amino acids K and L. It is noteworthy that cyclic annealing serves to mitigate this effect to a certain extent. Both the WAE and the RNN achieve high accuracy in reproducing the median of the peptide property distributions of the training data. In contrast, the LM samplers exhibit a clear preference for the basic amino acid R, resulting in a lower median of peptide properties compared to the training data. The median of the UniProt reference dataset is lower than that of the AMPs used for training, likely because the filters described in Sect. 2.1 removed potential AMPs from the UniProt dataset.

**Fig. 6:**
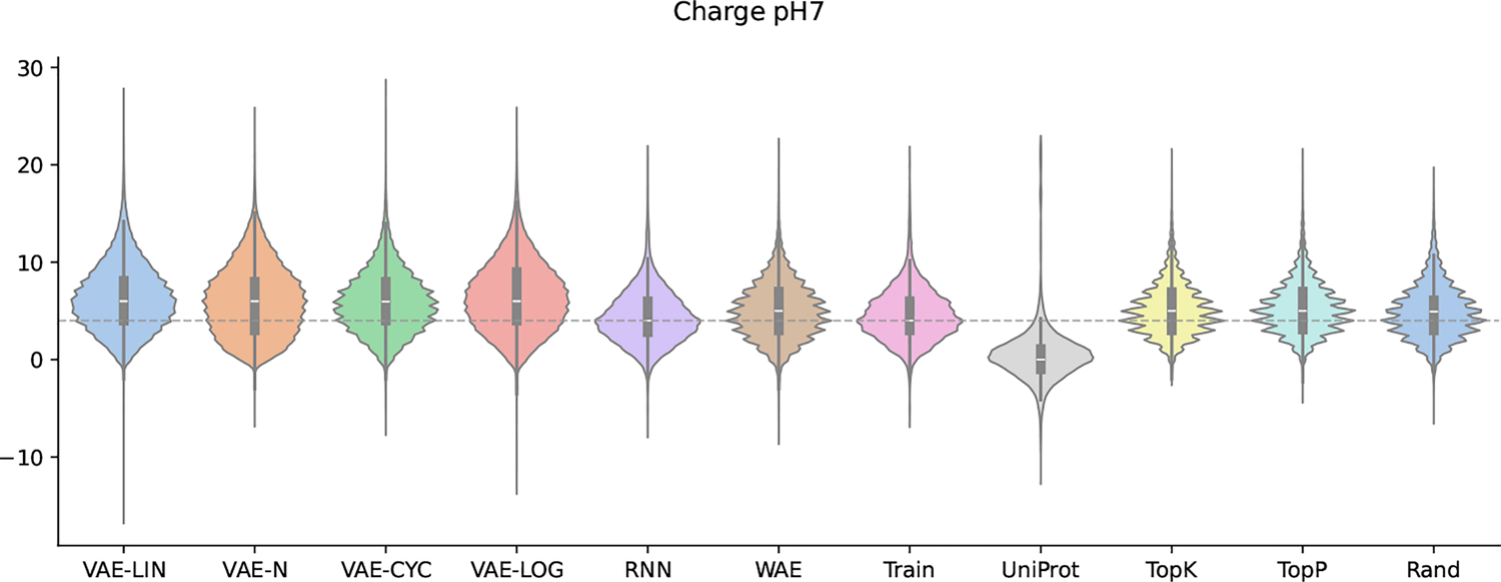
Distributions of the charge at pH 7. Distributions of the charge at pH 7 are shown as violin plots, including median and interquartile distances. The distributions of all template-free-generated sequences, as well as the training and comparison datasets, are shown. The gray dashed line marks the median of the training datasets’ distribution for easier visual comparison

Figure 6 shows the charge at pH 7 for the generated sequences, the training set, and the UniProt comparison dataset. The median line of the training set is shown as before in Fig. 4. The charge distributions of the generated sequences by the VAEs are all shifted slightly upwards compared to the training dataset. This can partly be attributed to an overrepresentation of the positively charged amino acid K.

The charge distribution of the sequences generated by the models RNN and WAE closely matches that of the training dataset, with the RNN reproducing the distribution almost perfectly. In the case of the WAE, the median is up-shifted by approximately one electron charge unit. As expected, the distribution of the UniProt dataset is shifted towards lower charge values because most peptides in this set are not expected to interact strongly with bacterial membranes. The distributions of the sequences generated by the different LM samplers are almost identical, with their medians slightly up-shifted compared to the training dataset.

Violin plots for the peptide properties isoelectric point, hydrophobicity, and length, together with the amino acid composition diagrams for VAE-L, VAE-N, VAE-LOG, and the Top-K and Top-P LM samplers, can be found in Supplementary Figures 1–5 .

**Table 1:** Summary presenting metrics for the evaluation of novelty, diversity, authenticity, and difference between peptide property distributions for template-free generated sequences. Subscripts indicate that this metric was calculated by comparing embeddings of training sequences

Dataset	Uniqueness	Diversity (10)	Novelty_train_	MMD_prop_	ATH_train_
UniProt	1.00	0.85	1.00	13.85	0.74
Helices	1.00	0.71	1.00	66.02	0.91
WAE	1.00	0.83	1.00	1.78	0.94
RNN	0.58	0.83	0.51	0.72	0.40
VAE-LIN	1.00	0.79	1.00	3.18	0.94
VAE-CYC	1.00	0.80	1.00	4.88	0.94
VAE-LOG	1.00	0.82	1.00	3.84	0.94
VAE-N	1.00	0.80	1.00	5.84	0.93
TOP-P	1.00	0.80	1.00	3.53	0.90
Top-K	1.00	0.80	1.00	2.37	0.92
Rand	1.00	0.82	1.00	1.82	0.90

As shown in Tab. 1, the metrics used to evaluate the novelty, diversity, authenticity, and property distribution disparities of generated peptides are outlined in comparison to the training data and reference datasets. Uniqueness was 1.00 across all datasets except the RNN-generated sequences (0.58). Diversity ranged from 0.71 (Helices) to 0.85 (UniProt). Among generated sequences, those from the RandomSampler and the WAE (Rand: 0.82, WAE: 0.83) exhibit the highest sequence diversity. As expected, the UniProt sequences showed the highest degree of diversity, while helices exhibited the least diversity. The remaining metrics for both datasets will not be addressed in this discussion.

Novelty was 1.00 across all datasets except for the RNN (0.51). The MMD_prop_ displayed notable variations across the generative models, with VAE-N (5.84) showing the largest deviation from the training distribution. WAE (1.78) and the RandomSampler (1.82) had the lowest MMD_prop_ values, indicating physicochemical properties most similar to the training data, while VAE-generated sequences showed slightly higher deviations (VAE-LIN: 3.18, VAE-LOG: 3.84, VAE-CYC: 4.88). Language model samplers (Top-K: 2.37, Top-P: 3.53) produced intermediate MMD_prop_ values. Authenticity was highest for sequences generated by the WAE and VAE models (around 0.94). Top-P (0.90) and RandomSampler (0.90) had slightly lower authenticity, while the RNN (0.40) performed poorly.

### Visualization of peptide feature space

Figure 4 presents a two-dimensional UMAP projection of feature vectors representing peptides’ physicochemical properties. The sequences generated by the WAE after filtering (Sect. 2.7 are represented in cyan, whereas the training dataset is shown in green.

The visualization reveals a continuous distribution of points, in contrast to the discrete, well-separated clusters that are often observed. Without multiple distinct clusters, the sequences form a cohesive and interconnected cloud. Within this central region, smaller subgroups or locally dense patches are evident, suggesting differences in sequence property profiles. These local density variations are distributed throughout the manifold without abrupt boundaries, indicating gradual transitions between peptide characteristics.

There are no substantial regions occupied exclusively by either the training data or the WAE-generated sequences. WAE-generated samples are consistently co-localized with training instances across the entire projection. In most regions containing training data points, WAE-generated samples appear in close spatial proximity. This suggests a close relationship between the two distributions at the resolution achieved by UMAP. The corresponding t-SNE visualization is provided in Supplementary Fig. 9.

### Template-based multi-sequence sampling

The results of the VAE multi-sampling process are presented in the following section. As mentioned in Sect. 2.6, 25 samples are generated for each template sequence. Here, a comparison is made between the generated samples and their respective templates to capture sequence similarity. Subsequently, the abundance of the generated variants is analyzed. The respective findings are summarized in Table 2.

To facilitate a comparison of the sequence similarity, the mean normalized Damerau-Levenshtein (LD) distance between each template sequence and its generated variants was calculated using the polars-ds library  [74]. The models VAE-LIN and VAE-CYC achieve the highest distances, with 0.526 and 0.512, respectively. The mean number of unique variants generated per template ranged from 13 to 14 for all VAEs. The application of different annealing schedules has been shown to reduce the number of posterior collapsed samples, as evidenced in Table 2.

Templates exhibiting posterior collapse are identified by producing only one particular sample. When an annealing schedule is applied, the number of templates for which at least 20 distinct variants can be generated increases. In this case, the VAE with the linear annealing schedule performs best.

**Table 2:** Summary presenting the mean normalized damerau-Levenshtein (LD) distance, the mean number of generated variants per template, the mean number of templates without distinct variants, and the number of templates with at least 20 variants

Auto-encoder	Mean Levenshtein distance	Mean # variants	# Collapses	$\geq$20
VAE-CYC	0.512	13	53	13
VAE-LIN	0.526	14	34	26
VAE-LOG	0.484	13	51	19
VAE-N	0.428	13	68	6

**Fig. 7:**
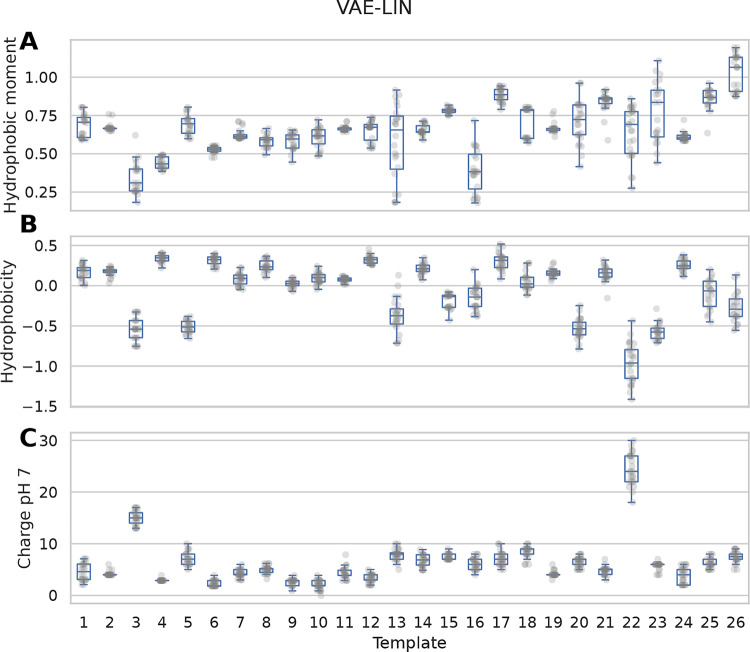
Descriptors of templates and the resulting variants of the VAE-L model. The hydrophobic moment (**A**), hydrophobicity (**B**), and charge at pH 7 (**C**) of the template and its generated sequences of the VAE with linear annealing. The transparent grey dots represent the single descriptor values for each generated variant of the templates 1–26, which are numbered on the x-axis

In order to compare the sequence properties of generated variants, the hydrophobicity, hydrophobic moment, and the charge at pH 7 were calculated using the peptides.py library and visualized in Fig. 7. For easier visualization, only templates with 20 or more generated variants from the VAE-LIN model were used in Fig. 7. Similar plots for the models VAE-N, VAE-LOG, and VAE-CYC are given in Supplementary Figures 6–8.

To illustrate the differences between the variants generated from template sequence GSKKPVPIIYCNRRTKCQRM, an example is given in Supplementary Fig. 10. This sequence was generated by the VAE model using the cyclic annealing schedule. In Supplementary Fig. 10, a multiple sequence alignment done with ClustalO  [75] and the biotite python library  [76] between the template sequence and the generated variants is displayed. Peptide positions with varying amino acids are shown with a lighter background color.

### Template-based sequence reconstruction

This section describes the results of the sequence reconstruction performed by the auto-encoder-based models. With each auto-encoder-based model, a single reconstruction sample was generated for all sequences from the reconstruction dataset. Table 3 presents the average reconstruction accuracy per peptide position and the number of fully reconstructed sequences.

**Table 3:** Mean reconstruction accuracy per peptide position and the number of fully reconstructed sequences for the auto-encoders

Model	Reconstruction accuracy	1:1 Reconstructions
WAE	69 %	111
VAE-CYC	60 %	25
VAE-LIN	57 %	20
VAE-LOG	56 %	10
VAE-N	51 %	5

**Fig. 8:**
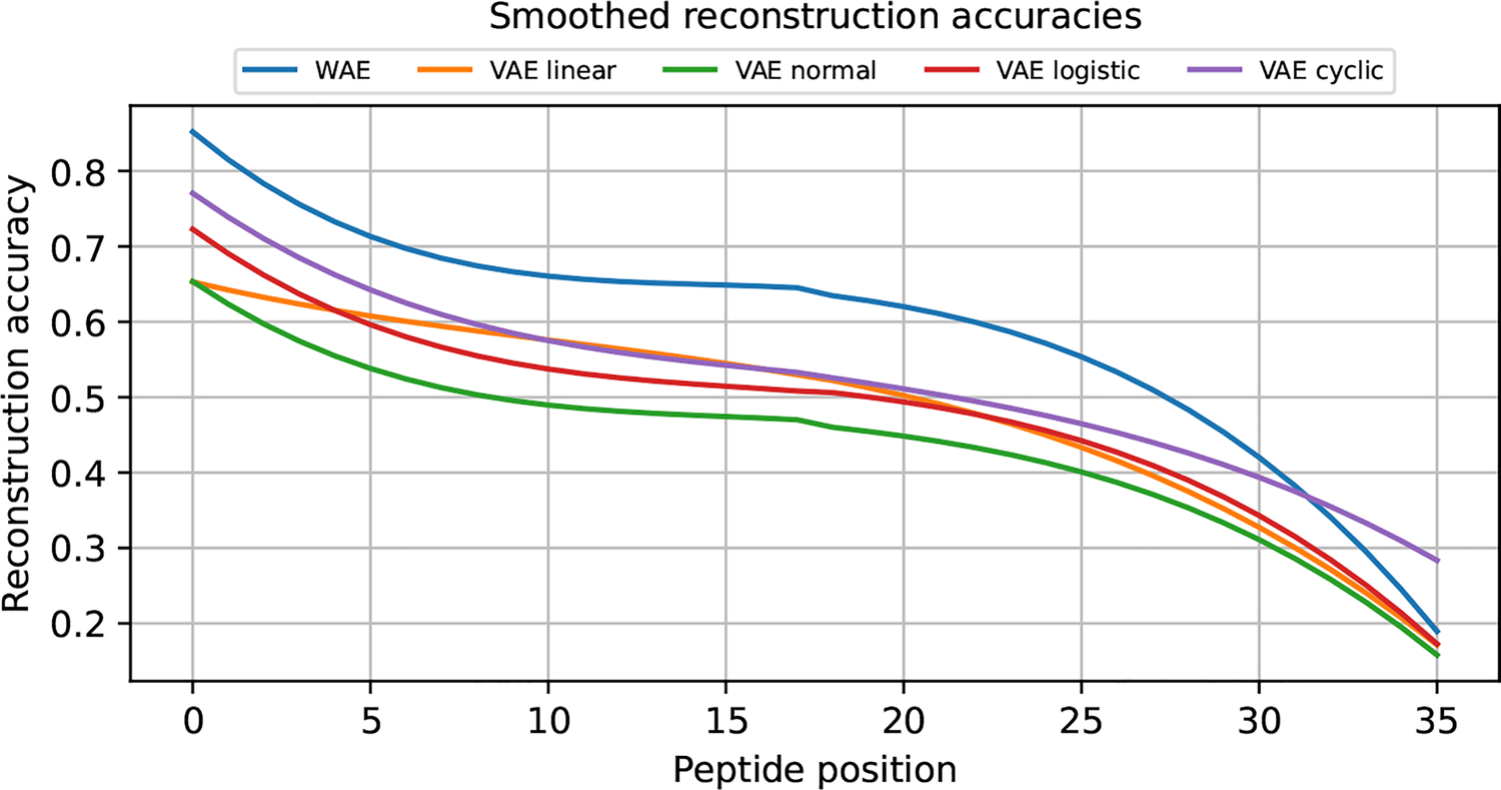
Sequence reconstruction accuracy smoothed by a Savitzky-Golay filter. Sequence reconstruction accuracy at each position for the five models. The accuracy of all models decreases with increasing peptide position. The WAE demonstrates high performance compared to the VAEs. Among the VAEs, VAE-CYC achieved the highest reconstruction accuracy

Figure 8 illustrates reconstruction accuracy across peptide positions per model. A Savitzky-Golay filter from the scipy library  [77] was used for smoothing. The WAE achieves the highest overall reconstruction accuracy compared to the VAEs, with a mean accuracy of 69 %. Furthermore, it produces the highest number of fully reconstructed peptides, with 111 of 720 identified. The VAE-N model performs the worst of all auto-encoders. The reconstruction accuracy achieved by the VAEs ranges from 51 % to 60 %, with a notable dependency on the annealing schedule employed. The VAE-CYC model exhibits the highest reconstruction accuracy, with a mean of 60 %.

### AMP prediction

This section is dedicated to the analysis of predictions concerning the potential activity of AMPs in template-free-generated sequences (see Table 4). The AMP prediction tools outlined in Sect. 2.8 were employed for this purpose.

In addition, sequences from the training dataset, as well as the comparison datasets described in Sect. 2.1, were incorporated into the analysis. Higher prediction rates are expected for the training and helices datasets, while the UniProt and RT datasets are expected to yield the lowest positive predictions.

**Table 4:** Predictions by AntiBP3, AMPScanner, Macrel, and AMPlify for all template-free generated datasets and the comparison datasets. The percentage of positive predictions with probability score $\geq$50 % and the majority vote of all tools are shown

Dataset	% AMPScanner	% AntiBP3	% Macrel	% AMPlify	% Majority
Train-AMPs	95.28	91.75	84.24	95.16	77.83
UniProt	36.56	5.98	5.84	11.1	0.79
Helices	95.46	91.56	98.18	91.95	85.04
RT	28.07	11.04	4.36	25.44	1.06
RSL	79.04	79.88	51.03	79.19	39.25
WAE	89.82	90.4	73.56	91.02	66.61
RNN	90.54	84.26	69.94	88.35	62.74
VAE-LIN	97.98	97.12	85.19	97.6	82.69
VAE-LOG	98.17	97.58	83.94	95.58	80.36
VAE-CYC	97.03	96.54	86.38	97.2	83.1
VAE-N	98.82	98.58	86.2	98.7	84.59
TOP-K	94.15	92.49	80.25	96.28	72.36
TOP-P	95.16	92.96	82.14	97.08	74.84
Rand	86.22	85.49	66.63	89.39	55.89

In the context of the training dataset, all tools demonstrated high prediction accuracy. AMPScanner (95.28 %) and AMPlify (95.16 %) performed nearly identically, while Macrel (84.24 %) exhibited the lowest sensitivity. The majority vote, which was predicted as AMPs by all four tools, yielded a 77.83 % consensus.

When evaluated on the UniProt dataset containing presumed non-AMPs, AntiBP3 (5.98 %) and Macrel (5.84 %) demonstrated the lowest rate of false predictions, while AMPScanner (36.56 %) exhibited a notably higher rate of misclassification. The majority vote for false predictions was found to be minimal (0.79 %). For the Helices dataset, all tools predicted AMP activity at rates comparable to the training data (Macrel: 98.18 %, AMPScanner: 95.46 %), with a majority vote of 85.04 %.

On totally randomly generated sequences (RT dataset), prediction rates declined substantially, with only 1.06 % of sequences predicted as AMPs by all tools. In contrast, the RSL dataset yielded high prediction rates along a higher majority vote compared to the RT dataset (39.25%).

Among the auto-encoder-based models, the VAEs achieved the highest prediction rates (97–98% for most tools), with VAE-N scoring the highest predictions. RNN-generated sequences demonstrated high prediction rates of approximately 84 % to 90 %, while the WAE exhibited lower predictions compared to the VAEs.

The prediction outcomes vary among the LM samplers. Top-K sampling generated sequences with high prediction rates (majority vote: 72.36 %). Similarly, Top-P sampling yielded sequences with slightly higher prediction rates (majority vote: 74.84 %) compared to Top-K. The RandomSampler, produced sequences with the lowest prediction rates (majority vote: 55.89 %). Additional metrics for the prediction are provided in Supplementary Tab. 8.

In addition to predicting active sequences from those generated using non-template-based sampling methods, the DBAASP database  [44] was searched for exact matches of the generated sequences. It was ensured that the generated sequences were not present in the training dataset and that only highly active AMPs annotated with a MIC value $\leq {10}\,{\mu}\textrm{M}$ were reported (Table 5). 

## Discussion

In this work, we compared different generative models, namely RNNs, VAEs, WAEs, and a tiny version of a language model, concerning their generative performance for antimicrobial peptides. Generated sequences were evaluated at the sequence and peptide-property levels.

### Sequence generation

The models for generating AMPs presented in this work all exhibit different advantages and disadvantages. For instance, VAEs facilitate the generation of multiple variants based on a single template, enabling the user to generate sequences from a specific AMP family of interest (Fig. 2). While the WAE can generate more diverse sequences using template-free sampling than VAE-based models (see Sect. 3.1), it cannot perform template-based multi-sequence sampling (see Sect. 2.6). Unlike VAE-based models, RNNs can only generate template-free sequences.

The RNN demonstrated satisfactory generative performance even without regularization (Figs. 5, 3, and 6). As shown by Müller et al. and confirmed in this study, RNNs can learn from relatively small datasets. Therefore, RNNs can be utilized in areas where experimentally validated peptide activity data is scarce. However, this model exhibits drawbacks in processing speed and tends to overfit when no proper regularization is used.

The LM employed in this study demonstrates medium generative performance, a phenomenon attributed to several factors. Usually, LM are trained on large-scale natural language datasets in combination with substantial pre-training, necessitating high-performance computing hardware and long training times  [60]. In contrast, the present study utilized a comparatively small language model with a limited dataset. For this reason, the capacity to capture all relevant information in the training data might be limited.

Interestingly, the model and training dataset are sufficiently large to achieve generative performance comparable to the VAE and WAE models (Figs. 5, 3, 6, and Tab. 1). The LM’s sampling strategies place considerable emphasis on amino acid frequencies. As shown by the amino acid composition comparison in Fig. 5, there is a clear over-preference for the amino acid arginine (R). Notably, this is one of the most frequently occurring amino acids in the training dataset, limiting the capacity for generating diverse sequences (see Table 1). This may be considered a potential drawback of this particular sampling strategy.

Additionally, the WAE reproduced the median of all the peptide descriptor distributions with the highest accuracy of all models. This is in line with the observation by Das et al.  [1], stating that the reconstructed sequences by the WAE were more diverse than those generated by a plain VAE. This observation should also hold for the template-free-generated sequences. Since, in contrast to VAEs, WAEs force the continuous mixture $Q_Z = \int Q(Z \mid X) dP_X$ to match the prior $P_{Z}$ as described in Sect. 2.4. As a results, latent representations of different sequences can stay farther away from each other than in the case of the VAE  [20]. This allows the WAE to generate higher-quality samples for both template-based and template-free generation.

As illustrated in Table 1, the high uniqueness and novelty scores (1.00) across the majority of the models demonstrate their ability to produce diverse and non-redundant sequences, except for the RNN, which generates many duplicate sequences (uniqueness = 0.58, novelty = 0.51). This suggests that recurrent architectures without regularization may struggle to explore beyond training data distributions in AMP generation tasks. This observation is consistent with the established limitations in capturing long-range dependencies.

With the exception of the RNN, the WAE and RandomSampler produced sequences with physicochemical properties closest to the training data (WAE: 1.78, Rand: 1.82). Among the VAE models, particularly VAE-N (5.84), demonstrated a slightly more property diversity, potentially offering a middle ground for controlled exploration. The Top-K and Top-P samplers achieved a balanced outcome, with Top-K generating more training-like sequences (2.37), while Top-P allowed slightly greater divergence (3.53), likely due to its dynamic sampling strategy. The findings depicted in Figs. 3 and 6 provide additional support for this assertion.

The Authenticity metric underscores that WAE and VAE models excel at generating highly plausible AMP-like sequences (approx. 0.94), whereas RNN sequences poorly matched the training distribution (0.40). This discrepancy may be attributed to the explicit modeling of latent distributions of AMPs by VAEs and WAEs.

The lower authenticity of sequences generated by the RandomSampler (0.90) compared to the other samplers, suggests that unconstrained randomness might sacrifice biological relevance. Among the generative models, WAE and the RandomSampler maintained high diversity while preserving authenticity, whereas RNN-generated sequences were both less unique and less diverse, highlighting limitations in their generative capacity. The VAEs generated slightly less diverse sequences (0.79–0.82) in comparison to the WAE and RandomSampler (Table 1).

It is conceivable that the set of template-free-generated sequences by the WAE might contain new unknown AMPs because the predictions made in Sect. 2.8 were not as high as the ones for the data generated by the VAEs. This hypothesis is further substantiated by the observation that the WAE-generated sequences exhibited a more substantial alignment with the distributions of peptide properties compared to those generated by the VAEs (see Figs. 6, 3, and Table 1).

Additionally, the analysis of the amino acid compositions between the training and generated sequences revealed a reduced disparity in the case of the WAE (see Fig. 5).

The VAEs also exhibited a pronounced prevalence for incorporating hydrophobic amino acids into the generated sequences (see Fig. 5). This tendency can contribute to an augmented hydrophobic moment and the preference for helical structures  [78]. These structural elements are likely to be more favorably predicted by the respective models, given their enhanced similarity to the training data of the individual models or to strong AMPs in general (see Tab. 4). A similar observation was reported by Müller et al.  [17].

However, additional research is required to validate the hypothesis that the WAE can generate novel and previously unidentified AMPs. Validation could include sequence analysis, motif enrichment, or evaluation of predictor blind spots and misclassification patterns.

### Insights from UMAP and t-SNE visualizations

Visualizing the generated samples or their properties can be helpful in the selection process. For auto-encoder-based models, this can be accomplished by a visualization of the latent representation by t-SNE or UMAP, as done previously by Renaud et al.  [27] and Dean et al.  [24, 25]. Alternatively, peptide properties can be used. In this study, feature vectors, i.e., peptide properties, are used rather than the auto-encoder’s latent representations. This approach enables the selection process to be guided in a more reference-based direction when template-free generation is employed.

In this case, known AMPs of interest serve as the comparison target. This is especially useful when WAE-based models are used for peptide generation, because, as outlined in Sect. 2.4, these models do not support template-based multi-sequence generation. Nonetheless, WAE models outperform VAEs at retaining relevant properties of the training data, as shown by Tolstikhin et al.  [20]. This is consistent with our findings on AMP properties (Figs. 3 and 6).

The absence of clear separation between the clustered instances in Fig. 4 and Supplementary Figures 9 and 13 supports the previous observation that the generated peptides exhibit high similarity in terms of peptide properties to the original training data. Additionally, the generated sequences show a high degree of diversity, as they can be found in almost all regions of the two-dimensional maps where the training data can be found.

Furthermore, comparing the t-SNE visualizations from WAE and VAE-N (Supplementary Figures. 9 and 13) with those from the RNN (Supplementary Fig. 12) confirms the expectation that the RNN is overfitting. In contrast, WAE and VAE-N exhibit a broader spread around the training data points, with more comprehensive coverage of empty regions. This suggests that these models are less prone to overfitting.

### AMP prediction

In silico prediction of the generated peptides activity should not be used as a sole indicator for candidate selection. Depending on the use case, a multi-level comparative approach should include peptide properties or sequence similarity to a set of reference peptides of interest. The results of the prediction task are indicative of both the strengths and limitations of the AMP prediction tools used in this work.

All four tools performed well on the training dataset, demonstrating strong generalization to known AMPs. However, their performance varied on presumed non-AMP sequences from the UniProt dataset. Notably, AMPScanner showed a higher number of false discoveries, suggesting that it may over-predict AMPs when applied to diverse peptide sequences.

In contrast, Macrel demonstrated the most conservative performance, exhibiting the lowest false-positive rate (5.84 %) but concomitantly displaying reduced sensitivity on certain datasets. These observations are in line with Santos-Júnior et al.  [36]. The majority voting indicated that, while individual tools may over-predict, their combined predictions can effectively filter out most non-AMPs.

A notable finding is the pronounced bias toward helical sequences, as all tools predicted the Helices dataset as AMPs at rates exceeding 90 %. Consequently, it can be hypothesized that the employed predictors may exhibit a preference for helical structural motifs, potentially resulting in the exclusion of non-helical AMPs that could be biologically active.

The RSL dataset, generated from the amino acid and length distributions of the training data, yielded high prediction rates, confirming that the tools may rely heavily on compositional features (e.g., charge and hydrophobicity) rather than on sequence novelty. This claim is further substantiated by the elevated prediction rates for VAE-generated sequences and their shifted peptide property distributions, as shown in Figs. 6 and 3.

The trade-off between predictability and diversity is critical for AMP discovery. Although VAEs and the RNN generate sequences with high prediction scores, their similarity to known AMPs may constrain the generation of novel sequences. In contrast, the WAE and the LM samplers yield more diverse sequences (see Table 1), but with lower prediction confidence. This underscores the importance of enhanced generative models that strike a balance between semantic similarity to known AMPs and exploration of novel sequence space.

The Top-K and Top-P samplers generated sequences with high prediction rates. This finding suggests that they produce sequences closely aligned with known AMP motifs by restricting sampling to high-probability tokens. This approach is useful for generating reliable and predictable AMP candidates. However, this sampling approach may limit the generation of novel candidates. Conversely, the RandomSampler introduced higher sequence variability, resulting in lower prediction rates (see Table 1). This approach may yield a greater potential for discovering novel AMP sequences that diverge from established patterns. This suggests that modifying the sampling strategy can govern the exploration-exploitation trade-off in AMP design: Top-K/Top-P for conservative optimization and random samplers for broader exploration of sequence space.

The majority vote was found to be an effective method for reducing false-positive results, as evidenced by the UniProt and RT datasets. In these cases, consensus predictions decreased to less than 1 %. This finding suggests that implementing ensemble approaches, which integrate multiple predictors, has the potential to enhance the reliability of large-scale AMP screening methodologies.

AMP prediction was conducted for sequences generated by the VAEs using template-based multi-sequence sampling, as outlined in Sect. 3.3. After deduplication, a Levenshtein distance threshold of five or less was applied between generated sequences and their respective templates. At least 96 % of the sequences were predicted to be active by at least 2 models, with a probability of 80 % or higher.

## Summary and conclusion

The present study demonstrates that putative new antimicrobial peptides can be generated using various deep learning models, including variational and Wasserstein auto-encoders  [20], recurrent neural networks, and language models. As expected, the effectiveness of the methods used for sequence generation is shown to be model-dependent. Each model possesses distinct strengths and weaknesses.

For instance, the Wasserstein auto-encoder exhibited superior accuracy in reconstructing known antimicrobial peptides (Table 3). However, it cannot generate multiple samples from a single template sequence, a capability exhibited by the variational auto-encoders employed in this study (see Sect. 2.6). Conversely, the recurrent neural network demonstrated notable efficacy, as previously reported  [17].

This architecture can operate with reduced data requirements compared to other deep learning models but exhibits comparatively lower processing speeds. In addition, auto-encoder-based models outperform the language model utilized in this study. VAEs and WAEs aim to accurately reconstruct the input while matching the latent distribution to the prior distribution. However, the LM used in this work may not be as effective as auto-encoders for generating short antimicrobial peptides to the same extent without further modifications.

A critical consideration is the intended use case of the generative model in combination with an appropriate sampling method. Determining whether template-based or template-free sequence generation is appropriate for the intended application is a critical decision that requires careful consideration. Template-based sequence generation may be applied when an active peptide with desired properties is used to generate sequences with properties similar to those of the template. On the other hand, template-free sequence generation is suitable for cases lacking lead peptides.

Another point to consider is the choice of the in silico methods used to predict AMP activity. A plethora of tools are available for the prediction of peptide activities  [79]. The models’ sensitivity and specificity depend on the datasets and peptide activity thresholds used for training. Therefore, a model that fits the use case has to be selected.

Furthermore, the models utilized in this study demonstrated an ability to preserve essential peptide properties (see Figs. 3 and 6) and amino acid sequence positions (see Fig. 5) in the generated sequences. This finding strengthens the hypothesis that our models cab generate putative sequences with characteristics similar to those of known AMPs (see Figs. 3, 6, 4, and Supplementary Fig. 9). Using Manifold learning methods such as UMAP, we could support our findings that WAE-based generative models can generate sequences with a high degree of diversity and while maintaining relevant AMP properties (Table 1 and Fig. 4).

Notably, all models employed in this study generated AMP sequences that were not utilized for training purposes. Some of these sequences are present in the DBAASP database. Among those we found AMPs with experimentally determined MIC values reported as less than $\leq {10}\,{\mu}\textrm{M}$ (Tab. 5), proving that our models can indeed generate highly active AMP sequences.

**Table 5:** Selection of the found entries of the template free generated sequences in the DBAASP database with a reported MIC value $\leq {10}\,{\mu}\textrm{M}$. The model, sequence, and a PubMed reference from the DBAASP are displayed. None of these sequence was used for training

Model	Sequence	Pubmed reference
Rand	RRWWWRWRW	33324358
RNN	SRSELIVHQR	22210491
RNN	GLLSVLGSVAKHVLPHVVPVIAEKL	10601876
Top-K	WWRWWRRRW	33324358
Top-P	KKLLKLLKLLL	37227046
VAE-N	GLLKKLLKKLLKKI	39141008

### Recommendations

This section contains a series of pragmatic suggestions for further research in this area, which are derived from the results of the present study.

#### Generative model selection

The selection of generative models should be based on the specific objectives of their AMP design task. Instead of presuming the existence of a universally optimal architecture, it is more beneficial to assess each architecture’s strengths and limitations. WAE-based and denoising diffusion models are particularly well-suited for projects that focus on the generation of diverse sets of novel AMP candidates.

VAEs paired with template-based sampling are better suited for generating sequences based on a known lead peptide. Recent work has demonstrated that integrated model architectures, including transformers combined with variational or Wasserstein auto-encoders, achieve strong performance, particularly when Bayesian optimization methods are applied to latent representations  [28, 33, 80].

#### Approaches for detecting overfitting

An overfitting model typically reproduces the training data and generates samples with minimal or no variation. For template-free generated sequences, overfitting can be assessed using metrics such as distribution discrepancies or embedding authenticity, as presented in Table 1. Furthermore, visualizations of latent representations or feature vectors using methods such as UMAP or t-SNE (Fig. 4 and Supplementary Figure 9, 12, and 13) provide additional support for this evaluation.

In template-based multi-sequence sampling with auto-encoders, the Levenshtein distance and the number of distinct or identical samples per template (Table 2) help identify overfitting. At the peptide property level, per-template visualization of these properties (Fig. 7) offers further evidence.

#### Generated data analysis

Analysis of generated data should prioritize interpretability and explainability in addition to performance metrics. Techniques such as visual comparisons of generated peptide property distributions with reference distributions and latent-space visualizations help elucidate the model’s learning process. Moreover, it is imperative to evaluate peptide properties that correspond to the intended use case.

Furthermore, some properties are aggregate proxies for AMP activity, e.g., hydrophobic moment, whereas others, such as molecular weight, are sensitive to peptide length and therefore prone to introducing length biases.

#### Choice of the right prediction models

The selection of prediction tools should be informed by prioritizing objectives such as estimating true antimicrobial activity, assessing overall “AMP-likeness,” or other downstream objectives.

To ascertain whether a prediction model is sensitive to selective sequence patterns or properties, it is necessary to systematically test them against well-curated experimental data and synthetic data featuring such patterns, e.g., in silico-designed amphipathic helices. Furthermore, combining various predictors and using majority voting for the resulting ensembles is known to increase overall prediction robustness.

#### Advanced sampling strategies

Recently, it has been shown that jointly structuring the latent space by training variational auto-encoders together with predictors for AMP activity or peptide properties increases the generative capabilities of these models. This approach can be further enhanced by applying Bayesian optimization strategies directly to the latent space or to reduced-dimensional versions thereof  [28, 33]. The integration of these approaches enables steering the generation towards peptides with desired properties, thereby reducing the number of wasted samples.

#### Practical applications

The generative models and evaluation metrics presented in this work provide integration points for AMP discovery pipelines. In the context of early-stage candidate screening, auto-encoder-based models (WAEs/VAEs) have demonstrated the ability to generate template-free candidates on a large scale. Quantitative sequence metrics (see Table 1), such as diversity and authenticity, coupled with comparisons of peptide properties against reference datasets, enable initial filtering of sequences with desired attributes.

In lead optimization, template-based, multi-sequence sampling with VAE-based models facilitates the generation of targeted analogs. Metrics for sequence similarity, including the Levenshtein distance, and discrepancy measures on peptide property distributions, such as the MMD, guide the design of sequence variants while retaining activity profiles.

Moreover, applying manifold learning methods, such as UMAP or t-SNE, enables the visual pre-selection of candidates that meet desired physicochemical properties compared to template sequences. Incorporating these methods at the upstream stage of experimental workflows further narrows the search space for active peptides, thereby prioritizing candidates with computationally validated physicochemical properties and predicted activity.

This approach has been shown to expedite the transition from in silico design to wet-lab testing  [1], thereby mitigating bottlenecks in high-throughput screening. In this context, experimental capacity frequently restricts the exploration of novel antimicrobial scaffolds.

## Limitations of this study

This study provides a comprehensive evaluation of generative models for AMP design. However, it has several limitations that should be considered when interpreting the results and planning future work.

First, the performance of the models depends on the quality and size of the training dataset. Publicly available AMP sequence data used to train the models is certainly subject to biases related to sequence representation, experimental conditions, and annotation accuracy. Additionally, these datasets do not fully capture the diversity of AMPs present in nature, hampering the models’ ability to generate novel and diverse sequences across the entire feature space.

Second, the evaluation of the generated sequences relied heavily on in silico predictions of antimicrobial activity and physicochemical properties. While these predictions offer valuable insights, they do not fully capture the complexity of AMP behavior in vivo. For instance, the models do not explicitly consider factors such as post-translational modifications, specific cellular targets, toxicity, solubility, or peptide stability.

The prediction of additional attributes, such as toxicity and hemolytic activity, can facilitate early selection of promising candidates. However, these analyses were not included in the present study due to the absence of an experimental validation component and because existing toxicity prediction models, while reasonably effective at classifying toxic versus non-toxic peptides, generally show lower performance when predicting inhibitory concentrations  [81–84].

In contrast, current models for antimicrobial peptide classification achieve higher reliability and consistency. Substantial progress has been made in developing and benchmarking toxicity prediction approaches, and ongoing methodological advancements are expected to further improve their robustness and practical applicability.

As recently pointed out by Brizuela et al.  [39], model complexity does not primarily determine predictive performance. Instead, the quality of the experimental training data is the main limitation, since classical machine learning approaches perform comparably to models using recent deep learning architectures  [85].

AMP activity assays are highly dependent on experimental conditions, including pH and ionic strength. However, many AMPs in publicly available databases lack these critical annotations. Consequently, direct comparison of measurements across different laboratories is challenging.

Third, the study focused only on a limited set of generative models. While these architectures are well established, other approaches, such as diffusion models and flow-matching methods, have become increasingly popular in generative AMP design. Nonetheless, variational auto-encoders were chosen for their well-known behavior, computational efficiency, and interpretability through lower-dimensional latent-space representations.

## Electronic supplementary material

Below is the link to the electronic supplementary material.

Supplementary Material 1

Supplementary Material 2

## Data Availability

All data and trained models are available on Zenodo. The code to train the models is available on GitHub. Zenodo: 10.5281/zenodo.17411954. GitHub: https://github.com/devshibe/amp-autencoders.

## List of abbreviations

AA: Amino Acid
AMP: Antimicrobial Peptide
AMR: Antimicrobial Resistance
ELBO: Evidence Lower Bound
GRU: Gated Recurrent Unit
IC: Inhibitory Concentration
LM: Language Model
LR: Learning Rate
LSTM: Long-Short-Term-Memory
MIC: Minimum Inhibitory Concentration
MMD: Maximum Mean Discrepancy
RNN: Recurrent Neural Network
t-SNE: t-distributed Stochastic Neighbor Embedding
UMAP: Uniform manifold approximation and projection
VAE: Variational Auto-Encoder
WAE: Wasserstein Auto-Encoder

## References

[1] Das P, Sercu T, Wadhawan K, Padhi I, Gehrmann S, Cipcigan F, et al. Accelerated antimicrobial discovery via deep generative models and molecular dynamics simulations. Nat Biomed Eng. 2021;5(6):613–23. 10.1038/s41551-021-00689-x. 33707779 10.1038/s41551-021-00689-x

[2] Naghavi M, Vollset SE, Ikuta KS, Swetschinski LR, Gray AP, Wool EE, et al. Global burden of bacterial antimicrobial resistance 1990–2021: a systematic analysis with forecasts to 2050. Lancet. 2024;404(10459):1199–226. https://linkinghub.elsevier.com/retrieve/pii/S0140673624018671. 39299261 10.1016/S0140-6736(24)01867-1 PMC11718157

[3] Naddaf M. 40 million deaths by 2050: toll of drug-resistant infections to rise by 70%. Nature. 2024;633(8031):747–48. 10.1038/d41586-024-03033-w. 39289575 10.1038/d41586-024-03033-w

[4] Rice L. Federal funding for the study of antimicrobial resistance in nosocomial pathogens: No ESKAPE. J Infect Dis. 2008;197(8):1079–81. 10.1086/533452. 18419525 10.1086/533452

[5] Kurbatfinski N, Kramer CN, Goodman SD, Bakaletz LO. ESKAPEE pathogens newly released from biofilm residence by a targeted monoclonal are sensitized to killing by traditional antibiotics. Front MicrobiolFront. Microbiol. 2023;14:1202215. 10.3389/fmicb.2023.1202215. 10.3389/fmicb.2023.1202215 PMC10410267 37564292

[6] Roque-Borda CA, Primo LMDG, Franzyk H, Hansen PR, Pavan FR. Recent advances in the development of antimicrobial peptides against ESKAPE pathogens. Heliyon. 2024;10(11):e31958. https://linkinghub.elsevier.com/retrieve/pii/S2405844024079891. 10.1016/j.heliyon.2024.e31958 PMC11167364 38868046

[7] Magana M, Sereti C, Ioannidis A, Mitchell CA, Ball AR, Magiorkinis E, et al. Options and limitations in clinical investigation of bacterial biofilms. Clin Microbiol Rev. 2018;31(3):e00084–16. 10.1128/CMR.00084-16. 10.1128/CMR.00084-16 PMC6056845 29618576

[8] Olivares J, Bernardini A, Garcia-Leon G, Corona F, B. Sanchez M, Martinez JL. The intrinsic resistome of bacterial pathogens. Front MicrobiolFront. Microbiol. 2013;4:103. 10.3389/fmicb.2013.00103. 10.3389/fmicb.2013.00103 PMC3639378 23641241

[9] Magana M, Pushpanathan M, Santos AL, Leanse L, Fernandez M, Ioannidis A, et al. The value of antimicrobial peptides in the age of resistance. Lancet Infect Dis. 2020;20(9):e216–30. 10.1016/S1473-3099(20)30327-3. 10.1016/S1473-3099(20)30327-3 32653070

[10] Browne K, Chakraborty S, Chen R, Willcox MD, Black DS, Walsh WR, et al. A new Era of antibiotics: the clinical potential of antimicrobial peptides. IJMS. 2020;21(19):7047. 10.3390/ijms21197047. 32987946 10.3390/ijms21197047 PMC7582481

[11] Fjell CD, Hiss JA, Hancock REW, Schneider G. Designing antimicrobial peptides: form follows function. Nat Rev Drug Discov. 2012;11(1):37–51. https://www.nature.com/articles/nrd3591. 10.1038/nrd3591 22173434

[12] Bucataru C, Ciobanasu C. Antimicrobial peptides: opportunities and challenges in overcoming resistance. Microbiological Res. 2024;286:127822. https://www.sciencedirect.com/science/article/pii/S0944501324002234. 10.1016/j.micres.2024.127822 38986182

[13] Brogden KA. Antimicrobial peptides: pore formers or metabolic inhibitors in bacteria? Nat Rev Microbiol. 2005;3(3):238–50. https://www.nature.com/articles/nrmicro1098. 15703760 10.1038/nrmicro1098

[14] Dijksteel GS, Ulrich MMW, Middelkoop E, Boekema BKHL. Review: lessons learned from clinical trials using antimicrobial peptides (AMPs). Front MicrobiolFront. Microbiol. 2021;12:616979. https://www.ncbi.nlm.nih.gov/pmc/articles/PMC7937881/. 10.3389/fmicb.2021.616979 PMC7937881 33692766

[15] Sani M-A, Separovic F. How Membrane-Active Peptides Get into Lipid Membranes. Acc Chem Res. 49(6):1130–38. 10.1021/acs.accounts.6b00074. 2016-06-21. 10.1021/acs.accounts.6b00074 27187572

[16] Ho Y-H, Shah P, Chen Y-W, Chen C-S. Systematic analysis of intracellular-targeting antimicrobial peptides, Bactenecin 7, hybrid of Pleurocidin and Dermaseptin, proline–arginine-rich peptide, and lactoferricin B, by using Escherichia coli proteome microarrays. Mol Cellular Proteomics. 2016,06;15(6):1837–47. https://www.ncbi.nlm.nih.gov/pmc/articles/PMC5083092/. 10.1074/mcp.M115.054999 PMC5083092 26902206

[17] Müller A, Hiss J, Schneider G. Recurrent neural network model for constructive peptide design. J Chem Inf Model. 2018;58(2):472–79. 10.1021/acs.jcim.7b00414. 29355319 10.1021/acs.jcim.7b00414

[18] Van Oort CM, Ferrell JB, Remington JM, Wshah S, Li J. AMPGAN v2: machine learning-guided design of antimicrobial peptides. J Chem Inf Model. 2021;61(5):2198–207. 10.1021/acs.jcim.0c01441. 33787250 10.1021/acs.jcim.0c01441 PMC8281497

[19] Tucs A, Tran DP, Yumoto A, Ito Y, Uzawa T, Tsuda K. Generating ampicillin-level antimicrobial peptides with activity-aware generative adversarial networks. ACS Omega. 2020;5(36):22847–51. 10.1021/acsomega.0c02088. 32954133 10.1021/acsomega.0c02088 PMC7495458

[20] Tolstikhin I, Bousquet O, Gelly S, Schoelkopf B Wasserstein Auto-Encoders (2019. http://arxiv.org/abs/1711.01558. ArXiv:1711.01558 [cs, stat.

[21] Chen Q, Yang C, Xie Y, Wang Y, Li X, Wang K, et al. GM-pep: A high efficiency strategy to de novo design functional peptide sequences. J Chem Inf Model. 2022;62(10):2617–29. 10.1021/acs.jcim.2c00089. 35533298 10.1021/acs.jcim.2c00089

[22] Das P, et al. PepCVAE: semi-supervised targeted design of antimicrobial peptide sequences. 2018. http://arxiv.org/abs/1810.07743.

[23] Pandi A, Adam D, Zare A, Trinh VT, Schaefer SL, Burt M, et al. Cell-free biosynthesis combined with deep learning accelerates de novo-development of antimicrobial peptides. Nat Commun. 2023;14(1):7197. https://www.nature.com/articles/s41467-023-42434-9. 37938588 10.1038/s41467-023-42434-9 PMC10632401

[24] Dean SN, Walper SA. Variational autoencoder for generation of antimicrobial peptides. ACS Omega. 2020;5(33):20746–54. 10.1021/acsomega.0c00442. 32875208 10.1021/acsomega.0c00442 PMC7450509

[25] Dean SN, Alvarez JAE, Zabetakis D, Walper SA, Malanoski AP. PepVAE: variational autoencoder framework for antimicrobial peptide generation and activity prediction. Front MicrobiolFront. Microbiol. 2021;12, 725727 (10.3389/fmicb.2021.725727. 10.3389/fmicb.2021.725727 PMC8515052 34659152

[26] Hawkins-Hooker A, Depardieu F, Baur S, Couairon G, Chen A, Bikard D, et al. Generating functional protein variants with variational autoencoders. PLoS Comput Biol. 2021;17(2):e1008736. 10.1371/journal.pcbi.1008736. 10.1371/journal.pcbi.1008736 PMC7946179 33635868

[27] Renaud S, Mansbach RA. Latent spaces for antimicrobial peptide design. Digit Discov. 2023;2(2):441–58. http://xlink.rsc.org/?DOI=D2DD00091A.

[28] Pikalyova K, Akhmetshin T, Orlov A, Haney EF, Akhoundsadegh N, You J, et al. Design of highly potent antibiofilm, antimicrobial peptides using explainable artificial intelligence. J Chem Inf Model. 2026;66(1):744–55. 10.1021/acs.jcim.5c01992. 41432371 10.1021/acs.jcim.5c01992

[29] Szymczak P, Możejko M, Grzegorzek T, Jurczak R, Bauer M, Neubauer D, et al. Discovering highly potent antimicrobial peptides with deep generative model HydrAMP. Nat Commun. 2023;14(1):1453. https://www.nature.com/articles/s41467-023-36994-z. 36922490 10.1038/s41467-023-36994-z PMC10017685

[30] Wang X, Tang J-Y, Sun J, Dorje S, Sun T-Q, Peng B, et al. ProT-Diff: a modularized and efficient strategy for De novo generation of antimicrobial peptide sequences by integrating protein language and diffusion models. Adv Sci. 2024;11(43):2406305. 10.1002/advs.202406305. 10.1002/advs.202406305 PMC11578372 39319609

[31] Chen T, Vure P, Pulugurta R, Chatterjee P. AMP-Diffusion: integrating latent diffusion with protein language models for antimicrobial peptide generation. 2024. 10.1101/2024.03.03.583201.

[32] Kong Z, et al. ProtFlow: flow matching-based protein sequence design with comprehensive protein semantic distribution learning and high-quality generation. 2026. 10.64898/2026.02.14.705870v1.

[33] Menard J, Mansbach RA. Low-dimensional semi-supervised latent Bayesian optimization for designing antimicrobial peptides. 2026. http://arxiv.org/abs/2510.17569. ArXiv:2510.17569.

[34] Vaswani A, et al. Attention is all You need. 2023. http://arxiv.org/abs/1706.03762. ArXiv:1706.03762 [cs.

[35] Wan F, De La Fuente-Nunez C. Mining for antimicrobial peptides in sequence space. Nat Biomed Eng. 2023;7(6):707–08. https://www.nature.com/articles/s41551-023-01027-z. 37095317 10.1038/s41551-023-01027-z PMC11537457

[36] Santos-Júnior CD, Pan S, Zhao X-M, Coelho LP. Macrel: antimicrobial peptide screening in genomes and metagenomes. PeerJ. 2020;8:e10555. https://peerj.com/articles/10555. 10.7717/peerj.10555 PMC7751412 33384902

[37] King AM, Zhang Z, Glassey E, Siuti P, Clardy J, Voigt CA. Systematic mining of the human microbiome identifies antimicrobial peptides with diverse activity spectra. Nat Microbiol. 2023;8(12):2420–34. https://www.nature.com/articles/s41564-023-01524-6. 37973865 10.1038/s41564-023-01524-6

[38] Maasch JRMA, Torres MDT, Melo MCR, De La Fuente-Nunez C. Molecular de-extinction of ancient antimicrobial peptides enabled by machine learning. Cell Host MicrobeCell Host Microbe. 2023;31(8):1260–74.e6. https://linkinghub.elsevier.com/retrieve/pii/S1931312823002962. 10.1016/j.chom.2023.07.001 PMC11625410 37516110

[39] Brizuela CA, Liu G, Stokes JM, De La Fuente-Nunez C. Ai methods for antimicrobial peptides: progress and challenges. Microb Biotechnol. 2025;18(1):e70072. 10.1111/1751-7915.70072. 10.1111/1751-7915.70072 PMC11702388 39754551

[40] Szymczak P, Zarzecki W, Wang J, Duan Y, Wang J, Coelho LP, et al. AI-Driven antimicrobial peptide discovery: mining and generation. Acc Chem Res. 2025;58(12):1831–46. 10.1021/acs.accounts.0c00594. 40459283 10.1021/acs.accounts.0c00594 PMC12177927

[41] Zhou X, Liu G, Cao S, Lv J. Deep learning for antimicrobial peptides: computational models and databases. J Chem Inf Model. 2025;65(4):1708–17. 10.1021/acs.jcim.5c00006. 39927895 10.1021/acs.jcim.5c00006

[42] Zhou X, Liu G, Han C, Lv J. ABPDB: a database of antibacterial peptides. IEEE Trans Comput Biol Bioinform. 2025;22(5):2300–06. https://ieeexplore.ieee.org/document/11048744/. 40811252 10.1109/TCBBIO.2025.3582844

[43] Gawde U, Chakraborty S, Waghu FH, Barai RS, Khanderkar A, Indraguru R, et al. CAMPR4: a database of natural and synthetic antimicrobial peptides. Nucleic Acids Res. 2023;51(D1):D377–83. 10.1093/nar/gkac933. 10.1093/nar/gkac933 PMC9825550 36370097

[44] Pirtskhalava M, Amstrong AA, Grigolava M, Chubinidze M, Alimbarashvili E, Vishnepolsky B, et al. DBAASP v3: database of antimicrobial/cytotoxic activity and structure of peptides as a resource for development of new therapeutics. Nucleic Acids Res. 2021;49(D1):D288–97. 10.1093/nar/gkaa991. 10.1093/nar/gkaa991 PMC7778994 33151284

[45] Jhong J-H, Yao L, Pang Y, Li Z, Chung C-R, Wang R, et al. dbAMP 2.0: updated resource for antimicrobial peptides with an enhanced scanning method for genomic and proteomic data. Nucleic Acids Res. 2022;50(D1):D460–70. 10.1093/nar/gkab1080. 10.1093/nar/gkab1080 PMC8690246 34850155

[46] Das D, Jaiswal M, Khan FN, Ahamad S, Kumar S. PlantPepDB: a manually curated plant peptide database. Sci Rep. 2020;10(1):2194. 10.1038/s41598-020-59165-2. 32042035 10.1038/s41598-020-59165-2 PMC7010657

[47] Wang G, Li X, Wang Z. APD3: the antimicrobial peptide database as a tool for research and education. Nucleic Acids Res. 2016;44(D1):D1087–93. 10.1093/nar/gkv1278. 10.1093/nar/gkv1278 PMC4702905 26602694

[48] Zhao X, Wu H, Lu H, Li G, Huang Q. LAMP: A Database Linking Antimicrobial Peptides. PLoS One. 2013;8(6):e66557. 10.1371/journal.pone.0066557. 10.1371/journal.pone.0066557 PMC3688957 23825543

[49] Bournez C, Riool M, de Boer L, Cordfunke RA, de Best L, van Leeuwen R, et al. CalcAMP: a new machine learning model for the accurate prediction of antimicrobial activity of peptides. Antibiot (Basel, Switz). 2023;12(4):725. 10.3390/antibiotics12040725. 10.3390/antibiotics12040725 PMC10135148 37107088

[50] Bateman A, Martin M-J, Orchard S, Magrane M, Ahmad S, Alpi E, et al.The UniProt Consortium. UniProt: the universal protein knowledgebase in 2023. Nucleic Acids Res. 2023;51(D1):D523–31. https://academic.oup.com/nar/article/51/D1/D523/6835362. 10.1093/nar/gkac1052 PMC9825514 36408920

[51] Watson M, et al. Kerashub. 2024. https://github.com/keras-team/keras-hub.

[52] Larralde M peptides.py (2022. https://github.com/althonos/peptides.py.

[53] Möller-Larsen R, et al. Seqme: a python library for evaluating biological sequence design. 2025. http://arxiv.org/abs/2511.04239. ArXiv:2511.04239.

[54] Olson D, Colligan T, Demekas D, Roddy JW, Youens-Clark K, Wheeler TJ. NEAR: neural embeddings for amino acid relationships. Bioinformatics. 2025;41(Supplement_1):i449–57. https://academic.oup.com/bioinformatics/article/41/Supplement_1/i449/8199346. 10.1093/bioinformatics/btaf198 PMC12261438 40662775

[55] Larralde MA. 2026. https://github.com/althonos/mininear.Original-date:2025-08-26T16:25:15Z.

[56] Kingma DP, Welling MA-EVB. 2022. http://arxiv.org/abs/1312.6114. ArXiv:1312.6114 [cs, stat.

[57] Gretton A, Borgwardt KM, Rasch MJ, Schölkopf B, Smola A. A kernel two-sample test. J Mach Learn Res. 2012;13:723–73.

[58] Hochreiter S, Schmidhuber J. Long short-term memory. Neural Computation. 1997;9(8):1735–80. 10.1162/neco.1997.9.8.1735. 9377276 10.1162/neco.1997.9.8.1735

[59] Cho K, van Merrienboer B, Bahdanau D, Bengio Y. On the properties of neural machine translation: encoder-decoder approaches. 2014. http://arxiv.org/abs/1409.1259. ArXiv:1409.1259 [cs, stat].

[60] Elnaggar A, Heinzinger M, Dallago C, Rehawi G, Wang Y, Jones L, et al. ProtTrans: toward understanding the language of life through self-supervised learning. IEEE Trans Pattern Anal Mach Intell. 2022;44(10):7112–27. https://ieeexplore.ieee.org/document/9477085/. 34232869 10.1109/TPAMI.2021.3095381

[61] Brandes N, Ofer D, Peleg Y, Rappoport N, Linial M. ProteinBERT: a universal deep-learning model of protein sequence and function. Bioinformatics. 2022;38(8):2102–10. https://academic.oup.com/bioinformatics/article/38/8/2102/6502274. 35020807 10.1093/bioinformatics/btac020 PMC9386727

[62] Ferruz N, Schmidt S, Höcker B. ProtGPT2 is a deep unsupervised language model for protein design. Nat Commun. 2022;13(1):4348. https://www.nature.com/articles/s41467-022-32007-7. 35896542 10.1038/s41467-022-32007-7 PMC9329459

[63] Chollet F, et al. Keras. 2015. https://keras.io.

[64] Lucas J, Tucker G, Grosse R, Norouzi M. Don’t blame the ELBO! A Linear VAE Perspective on Posterior Collapse. 2019. http://arxiv.org/abs/1911.02469. ArXiv:1911.02469.

[65] Fu H, et al. Cyclical annealing schedule: a simple approach to mitigating KL vanishing. 2019. http://arxiv.org/abs/1903.10145. ArXiv:1903.10145 [cs, stat.

[66] Veltri D, Kamath U, Shehu A. Deep learning improves antimicrobial peptide recognition. *Bioinformatics (Oxford, England)*. Bioinformatics. 2018;34(16):2740–47. 10.1093/bioinformatics/bty179. 29590297 10.1093/bioinformatics/bty179 PMC6084614

[67] Bajiya N, Choudhury S, Dhall A, Raghava GPS. Antibp3: a method for predicting antibacterial peptides against gram-positive/negative/variable bacteria. Antibiotics. 2024;13(2):168. https://www.ncbi.nlm.nih.gov/pmc/articles/PMC10885866/. 38391554 10.3390/antibiotics13020168 PMC10885866

[68] Li C, Sutherland D, Hammond SA, Yang C, Taho F, Bergman L, et al. Amplify: attentive deep learning model for discovery of novel antimicrobial peptides effective against WHO priority pathogens. BMC Genomics. 2022;23(1). 10.1186/s12864-022-08310-4. 10.1186/s12864-022-08310-4 PMC8788131 35078402

[69] McInnes L, Healy J, Saul N, Großberger L. UMAP: uniform manifold approximation and projection for dimension reduction. ArXiv e-prints. J Educ Chang Open Source Softw. 2018;3(29):861. 10.21105/joss.00861.

[70] Maaten LVD, Hinton G. Visualizing data using t-sne. J Mach Learn Res. 2008;9:2579–605.

[71] McInnes L, Healy J, Saul N, Grossberger L. Umap: uniform manifold approximation and projection. JOSS. 2018;3(29):861. 10.21105/joss.00861.

[72] Poličar PG, Stražar M, Zupan B. Opentsne: a modular python library for t-sne dimensionality reduction and embedding. J Stat Soft. 2024;109(3):1–30. https://www.jstatsoft.org/index.php/jss/article/view/v109i03.

[73] Bokeh Development Team. Bokeh: python library for interactive visualization. 2025. https://bokeh.org/.

[74] Qin T. Polars for data science. 2024. https://gtihub.com/abstractqqq/polars_ds_extension.

[75] Sievers F, Higgins DG. Clustal Omega. CP Bioinf. 2014;48(1). 10.1002/0471250953.bi0313s48. 10.1002/0471250953.bi0313s48 25501942

[76] Kunzmann P, Hamacher K. Biotite: a unifying open source computational biology framework in python. BMC Bioinf. 2018;19(1):346. 10.1186/s12859-018-2367-z. 10.1186/s12859-018-2367-z PMC6167853 30285630

[77] Virtanen P, Gommers R, Oliphant TE, Haberland M, Reddy T, Cournapeau D, et al. SciPy 1.0: fundamental algorithms for scientific computing in python. Nat Methods. 2020;17(3):261–72. 10.1038/s41592-019-0686-2. 32015543 10.1038/s41592-019-0686-2 PMC7056644

[78] Eisenberg D, Weiss RM, Terwilliger TC. The hydrophobic moment detects periodicity in protein hydrophobicity. Proceedings of the National Academy of Sciences of the United States of America. 1984, 140–44 81. 10.1073/pnas.81.1.140 PMC344626 6582470

[79] Bárcenas O, Pintado-Grima C, Sidorczuk K, Teufel F, Nielsen H, Ventura S, et al. The dynamic landscape of peptide activity prediction. Comput Struct Biotechnol J. 2022;20:6526–33. https://www.sciencedirect.com/science/article/pii/S2001037022005384. 36467580 10.1016/j.csbj.2022.11.043 PMC9712827

[80] Sevgen E, et al. ProT-VAE: protein transformer variational AutoEncoder for functional protein design. Proceedings of the National Academy of Sciences. 2025, e2408737122). doi: 10.1073/pnas.2408737122 122. 10.1073/pnas.2408737122 PMC12541330 41052325

[81] Rathore AS, Choudhury S, Arora A, Tijare P, Raghava GPS. ToxinPred 3.0: an improved method for predicting the toxicity of peptides. Comput Biol Med. 2024;179:108926. https://www.sciencedirect.com/science/article/pii/S0010482524010114. 39038391 10.1016/j.compbiomed.2024.108926

[82] Qiu P, Feng H, Zhang M-C, Poczos B. AmpLyze: a deep learning model for predicting the hemolytic concentration. 2025. http://arxiv.org/abs/2507.08162. ArXiv:2507.08162.

[83] Wang J-H, Sung T-Y. ToxTeller: predicting peptide toxicity using four different machine learning approaches. ACS Omega. 2024;9(29):32116–23. 10.1021/acsomega.4c04246. 39072096 10.1021/acsomega.4c04246 PMC11270677

[84] Rathore AS, Kumar N, Choudhury S, Mehta NK, Raghava GPS. Prediction of hemolytic peptides and their hemolytic concentration. Commun Biol. 2025;8(1):176. https://www.ncbi.nlm.nih.gov/pmc/articles/PMC11794569/. 39905233 10.1038/s42003-025-07615-w PMC11794569

[85] García-Jacas CR, Pinacho-Castellanos SA, García-González LA, Brizuela CA. Do deep learning models make a difference in the identification of antimicrobial peptides? Briefings Bioinf. 2022;23(3):bbac094. 10.1093/bib/bbac094. 10.1093/bib/bbac094 35380616

