# Supplementary material for: Generative models for antimicrobial peptide design: auto-encoders and beyond
Source: BioData Min. 2026 May 9;19:42. doi: 10.1186/s13040-026-00558-w (PMC13181977; doi:10.1186/s13040-026-00558-w)
Supplement: Supplementary file 2 — Supplementary Material 2 [file 13040_2026_558_MOESM2_ESM.pdf]

Generative Models for Antimicrobial Peptide Design: Auto-Encoders and Beyond

Author names:

Lukas Beierle[1*], Julian M. Hahnfeld[1], Alexander Goesmann[1], Reihaneh Mostolizadeh[1,3], Franz Cemič[2]

Affiliation:

[1] Bioinformatics and Systems Biology, Justus Liebig University Giessen, Giessen, Hesse, Germany.
[2] Department of Computer Science, University of Applied Sciences Giessen, Giessen, Hesse, Germany.
[3] Max-Planck-Institute for Terrestrial Microbiology, Marburg, Hesse, Germany

* Corresponding author:

E-mail: lukas.beierle@computational.bio.uni-giessen.de

Keywords:

antimicrobial peptides, generative models, deep learning, variational auto-encoders, wasserstein auto-encoder, language models

URLs:

- Zenodo
- GitHub

Hardware and operating system

The models were trained on a Dell OptiPlex 7090 computer with an Intel Core i7-11700, 32 gigabytes of DDR4 RAM, and an NVIDIA GeForce GTX 1660 Super graphics card. As an operating system, the Linux distribution OpenSUSE Tumbleweed was used.

Supplemental Tables

Table 1: Versions of the python libraries used for the implementation of the generative models and the visualization of generated peptide sequences. For the implementation of the language model some of the libraries were used with different versions, mainly due to the keras_hub library.

Library	Version	Used for
**Python**	3.11.7	Programming
**Polars**	0.20.10	Dataframes and statistics (VAEs)
**Polars**	>= 1.0.0	Language model evaluation
**Keras**	2.15.0	Deep learning models
**Keras**	>= 3.0.0	Language model implementation
**KerasHub**	>= 0.18.0	Language model implementation
**Tensorflow**	2.15.0	VAE model implementation
**Tensorflow-gpu**	2.15.0	GPU support
**Tensorflow**	>= 2.18.0	Language model implementation
**tqdm**	4.66.2	Progress bars
**matplotlib**	3.8.2	Visualization
**Seaborn**	0.13.1	Visualization
**Numpy**	1.26.3	Required by Keras and Tensorflow
**peptides**	0.3.4	peptide properties
**Biotite**	>=1.0.0	Multiple sequence alignment

Table 2: Antimicrobial peptide source databases with the year of the last access or state of the database.

Database	Last state
**CAMP**	2023
**DBAASP**	2023
**APD3**	2023
**LAMP**	from Bournez et al. 2023
**dbamp**	2022
**PlantPepDB**	2020

Table 3: Overview of the hyper parameters: batch size, learning rate, number of epochs and latent dimension size used by the generative models.

Model	Batch size	Epochs	LR	Latent dimension
**WAE**	128	1.000	0.001	128
**VAE**	64	350	0.001	64
**RNN**	128	100	0.01	-
**LM**	64	100	0.001	-

Table 4: Mean, maximum and minimum percent differences in amino acid composition between training and randomly generated sequences. The amino acid with the highest deviance is also reported.

Model	Mean	Max	Min	Most deviating amino acid
**WAE**	-8.7	40.5	-54.9	Cysteine (C)
**VAE-CYC**	-19.4	66.0	-74.0	Tyrosine (Y)
**VAE-LIN**	-29.6	51.0	-91.7	Glutamic acid (E)
**VAE-LOG**	-18.5	72.8	-86.3	Methionine (M)
**VAE-N**	-27.9	60.0	-92.7	Glutamine (Q)
**RNN**	1.8	24.0	-14.8	Proline (P)
**Random**	-1.8	40.2	-23.5	Methionine (M)
**TopP**	-25.8	56.4	-92.1	Methionine (M)
**TopK**	-24.2	42.0	-95.7	Methionine (M)

Table 5: Full list of peptide properties that were calculated for the feature vectors used for UMAP and t-SNE and the Prop-MMD calculations.

Property	UMAP / t-SNE	Prop-MMD
Aliphatic index	✓	✓
Boman index	✓	✓
Charge	✓	✓
Hydrophobic moment	✓	✓
Hydrophobicity	✓	✓
Isoelectric point	✓	✓
Instability index	✓	✓
Molecular weight	✓	✓
Atchley factors	✓	✗
Fasgai vectors	✓	✗
VHSE scales	✓	✗
Physical descriptors	✓	✗
Kidera factors	✓	✗

Table 6: Mean, maximum and minimum percent differences in amino acid composition between training and randomly generated sequences. The amino acid with the highest deviance is also reported.

Algorithm	Parameter	Value
**UMAP**	n_neighbors	50
**UMAP**	random_state	42
**UMAP**	n_components	2
**t-SNE**	random_state	42
**t-SNE**	n_components	2
**t-SNE**	perplexity	100
**t-SNE**	negative_gradient_method	fft
**t-SNE**	initialization	pca
**t-SNE**	metric	cosine

Supplemental Figures

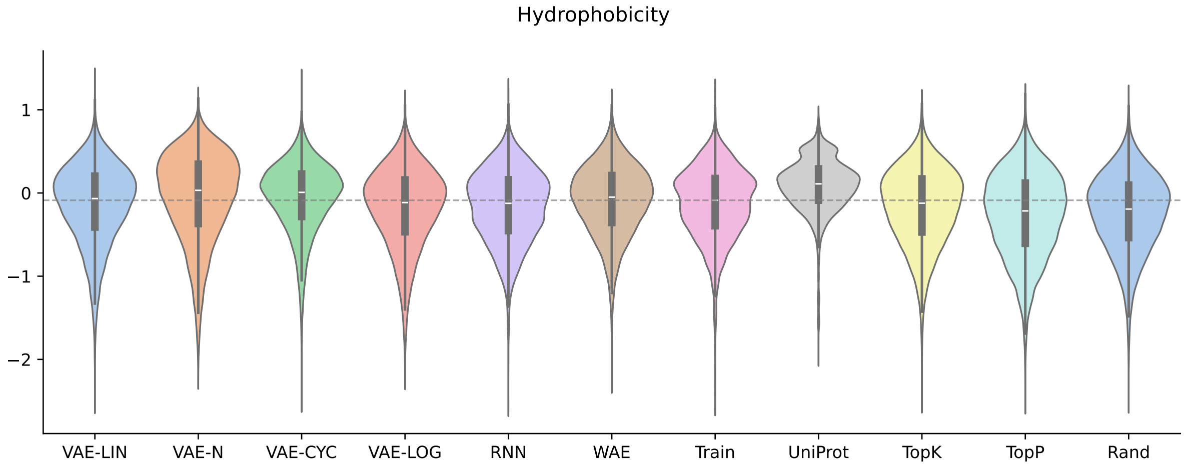

Figure 1: Distributions of the hydrophobicity shown as violin plots including median and interquartile distances. The distributions of all randomly generated sequences, as well as training and comparison data sets are shown. The grey dashed line marks the median of the training datasets' distribution for easier visual comparison.

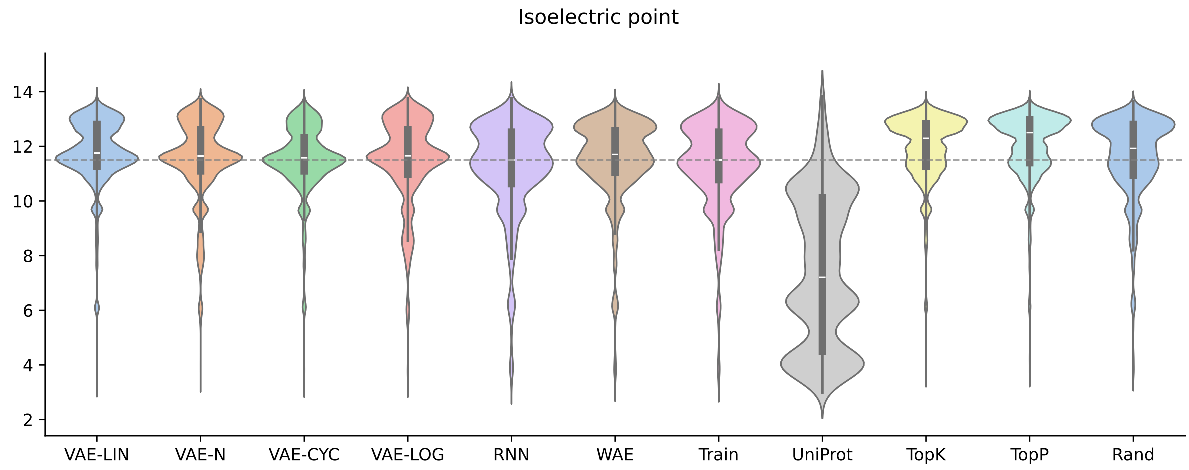

Figure 2: Distributions of the isoelectric point shown as violin plots including median and interquartile distances. The distributions of all randomly generated sequences, as well as training and comparison data sets are shown. The grey dashed line marks the median of the training datasets' distribution for easier visual comparison.

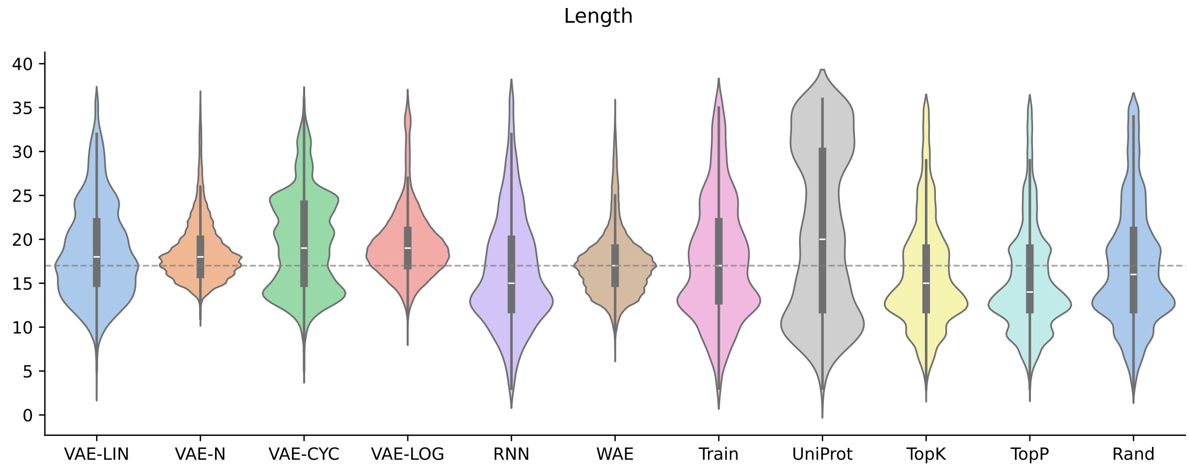

Figure 3: Distributions of the peptide length shown as violin plots including median and interquartile distances. The distributions of all randomly generated sequences, as well as training and comparison data sets are shown. The grey dashed line marks the median of the training datasets' distribution for easier visual comparison.

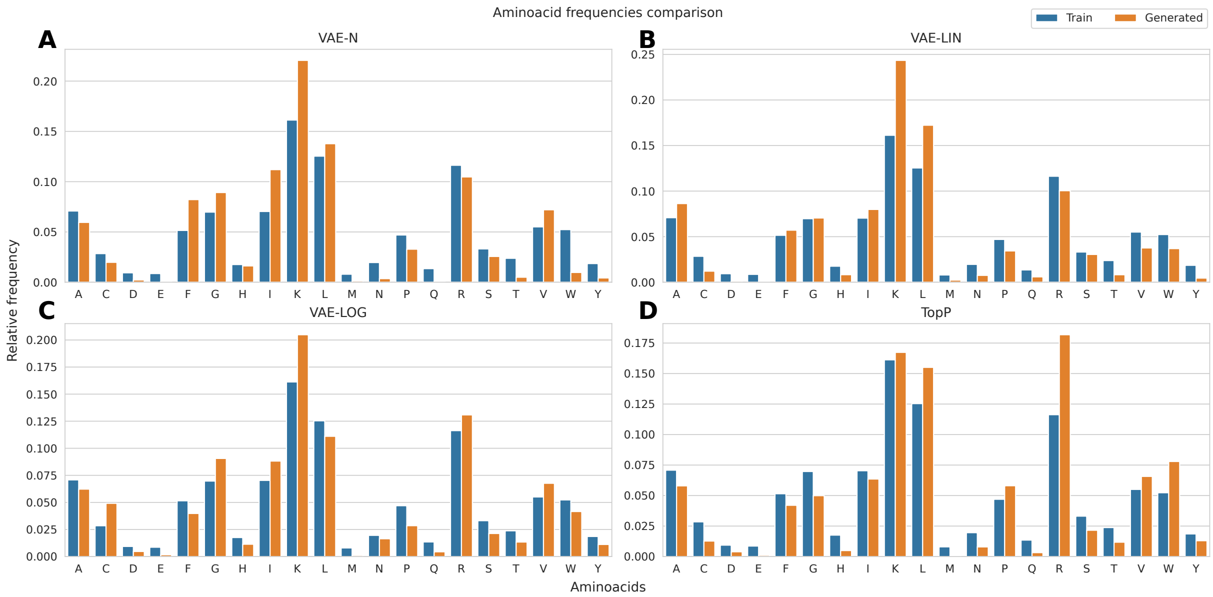

Figure 4: Amino acids comparison between the training set and generated sequences. Each of the sub figures shows the relative frequency of individual amino acids within the training sets (blue) and various generated sequences (orange). The results for the VAE without annealing, VAE with linear annealing, VAE with logistic annealing and TopPSampler are presented from the upper left to the lower right.

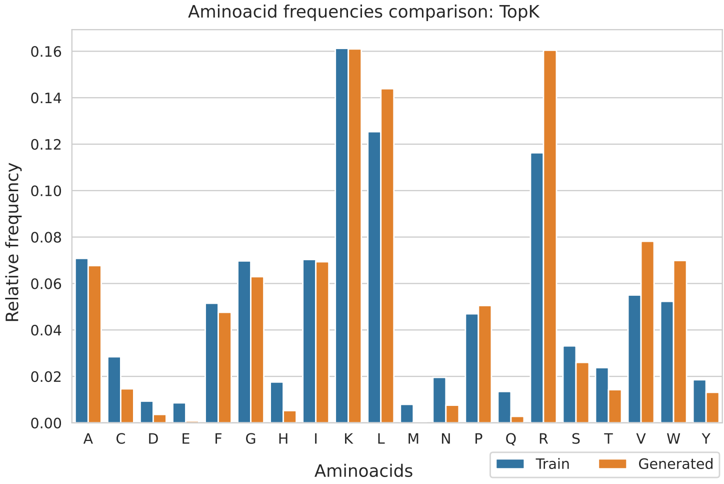

Figure 5: Amino acids comparison between the training set and the generated sequences of the TopK sampler. The relative frequency of individual amino acids within the training set (blue) and generated sequences (orange).

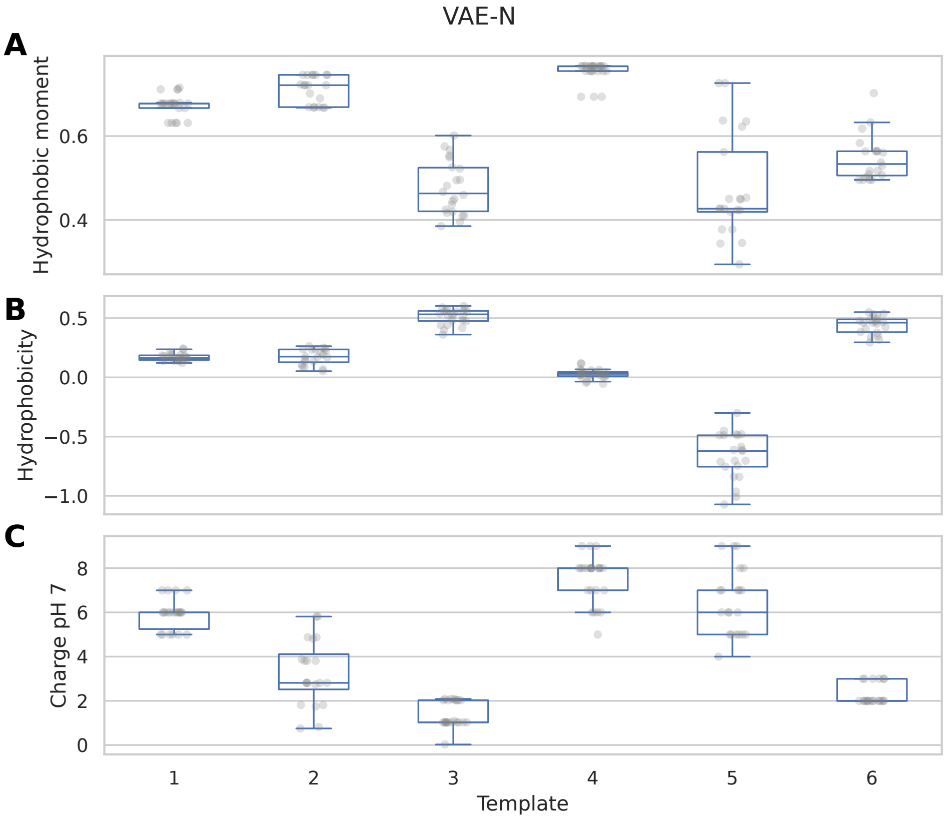

Figure 6: The hydrophobic moment, hydrophobicity and charge at pH 7 of the template and its generated sequences of the VAE without annealing. The transparent grey dots represent the single descriptor values for each generated variant of the templates 1-6, which are numbered on the x-axis.

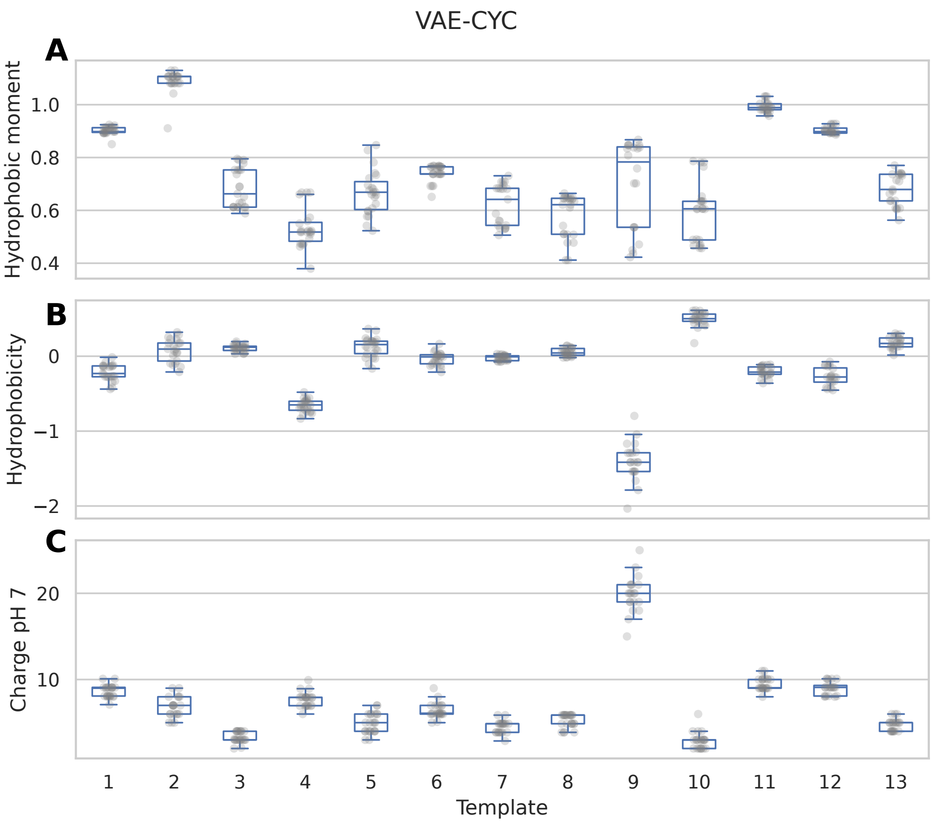

Figure 7: The hydrophobic moment, hydrophobicity and charge at pH 7 of the template and its generated sequences of the VAE with cyclic annealing. The transparent grey dots represent the single descriptor values for each generated variant of the templates 1-13, which are numbered on the x-axis.

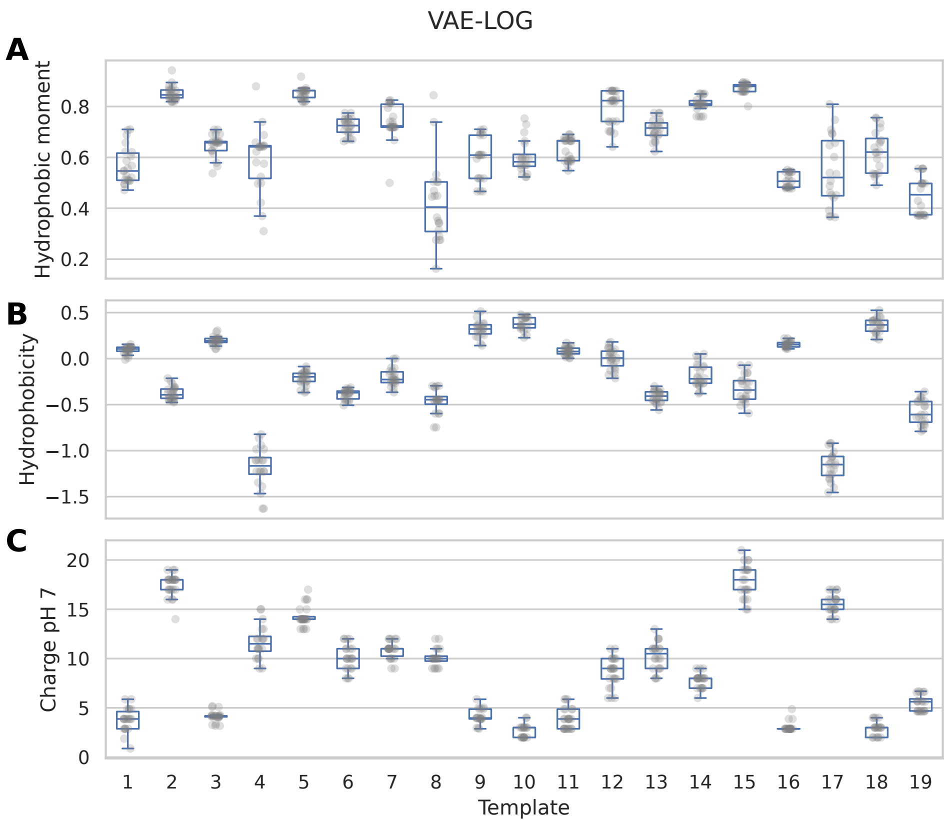

Figure 8: The hydrophobic moment, hydrophobicity and charge at pH 7 of the template and its generated sequences of the VAE with logistic annealing. The transparent grey dots represent the single descriptor values for each generated variant of the templates 1-29, which are numbered on the x-axis.

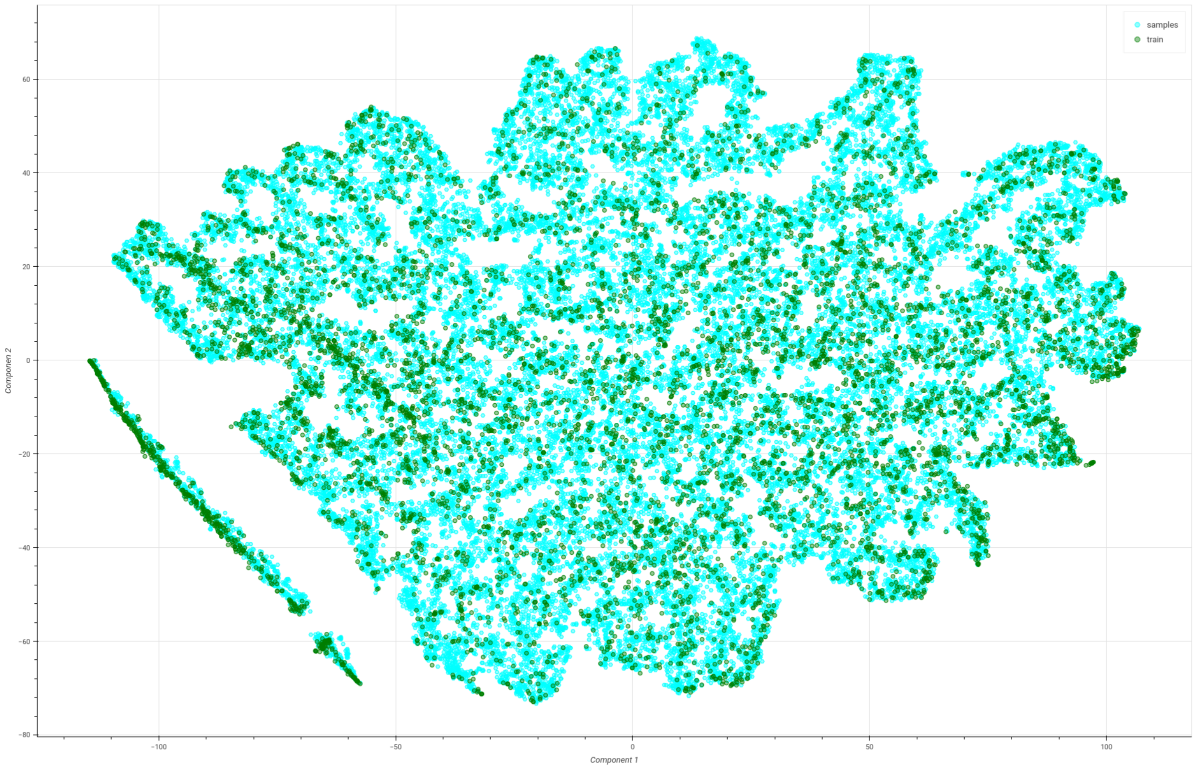

Figure 9: Visualization of the 2D representations created using t-SNE for sequences generated by the WAE.

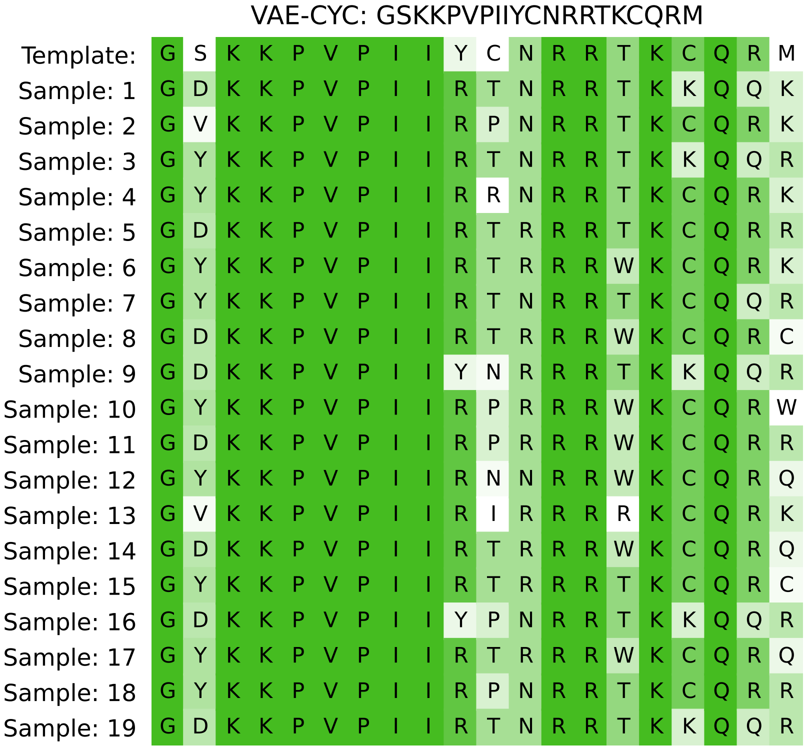

Figure 10: Visualization of an multiple sequence alignment from a single template sequence and the generated variants Multiple sequence alignment from the template sequence: GSKKPVPIIYCNRRTKCQRM and the generated variants by the VAE with cyclic annealing. The color corresponds to the number of variations in amino acids on the respective position in the peptide.

WAE and LM Hyperparamter Sensitivity

Several latent spaces were evaluated for the WAE model, specifically with dimensions of 32, 64, 128, and 256. Two model configurations were tested: LLM-s, the basic configuration, and LLM-l, a larger version with each parameter doubled. The number of layers varied from 1 to 4, as indicated by indices. All experiments used the same metrics as in the main manuscript.
Overall, there was no clear trend showing that either the smaller or larger configuration consistently outperformed the other.

		Charge (pH7)	HM	HP	Uniqueness	Novelty	ATH	Prop-MMD	Diversity
**llm-s**	**1**	4.89±0.03	0.54±0.01	-0.27±0.01	0.99	0.99	0.90	2.28	0.83
**llm-s**	**2**	4.98±0.03	0.59±0.01	-0.27±0.01	0.98	0.98	0.88	1.92	0.82
**llm-s**	**3**	4.72±0.03	0.58±0.01	-0.23±0.01	0.98	0.98	0.88	1.68	0.83
**llm-s**	**4**	4.68±0.03	0.59±0.01	-0.21±0.01	0.98	0.98	0.88	1.49	0.83
**llm-l**	**1**	4.86±0.03	0.57±0.01	-0.19±0.01	0.99	0.99	0.90	1.44	0.83
**llm-l**	**2**	4.55±0.03	0.59±0.01	-0.22±0.01	0.98	0.97	0.87	1.18	0.83
**llm-l**	**3**	4.72±0.03	0.60±0.01	-0.21±0.01	0.98	0.98	0.89	1.39	0.83
**llm-l**	**4**	4.75±0.03	0.60±0.01	-0.21±0.01	0.96	0.96	0.86	1.28	0.83
**wae**	**1**	5.03±0.04	0.57±0.01	-0.10±0.01	1.00	1.00	0.94	2.05	0.84
**wae**	**2**	5.07±0.03	0.61±0.01	-0.07±0.01	1.00	1.00	0.94	1.65	0.84
**wae**	**3**	5.05±0.03	0.60±0.01	-0.09±0.01	1.00	1.00	0.93	2.51	0.83
**wae**	**4**	5.83±0.05	0.65±0.01	-0.07±0.01	1.00	1.00	0.94	2.02	0.82
**train**	**1**	4.59±0.03	0.60±0.01	-0.13±0.01	1.00	0.00	0.00	0.00	0.83

Figure 11: Metrics for the different hyperparameter configurations for WAE and LMs. Included metrics are, charge at pH 7, hydrophobic moment, hydrophobic, hydrophobicity, uniqueness, novelty, authenticity, MMD on peptide properties and diversity.

Amino acids distribution Wasserstein distances

Table 7: Wasserstein distances between the amino acid distribution of random generated sequences and the training dataset.

Dataset	Wasserstein distance (Train)
**WAE**	0.0046
**VAE-LIN**	0.0143
**VAE-N**	0.0146
**VAE-CYC**	0.014
**VAE-LOG**	0.0081
**RNN**	0.003
**Top-P**	0.0109
**Top-K**	0.0096
**Rand**	0.004

Additional prediction metrics

Further baseline validation was conducted to assess the ability of the AMP prediction tools to distinguish between known AMPs (training dataset) and presumed non-AMPs (UniProt sequences). While this analysis does not address generalization performance, particularly given the potential overlap between the positive set and the tools' original training data, it shows that the predictors can reliably differentiate between well-defined AMP and non-AMP classes under controlled conditions. Based on these assumptions, commonly used classification metrics were calculated.

- Accuracy (ACC)
- Sensitivity (SEN)
- Specificity (SPC)
- Precision (PRE)

Table 8: Additional prediction metrics calculated using the training and UniProt dataset.

Model	TP	FP	TN	FN	ACC	SEN	SPC	PRE	F1
**AMPlify**	7251	369	492	3942	0.64	0.65	0.57	0.95	0.77
**AMPscanner**	7260	360	1621	2813	0.74	0.72	0.82	0.95	0.82
**AntiBP3**	6991	323	265	4169	0.62	0.63	0.45	0.96	0.76
**Macrel**	6419	1201	259	4175	0.55	0.61	0.18	0.84	0.7

Stability analysis for the WAE-generated sequences

The set of sequence generated by the WAE was split into 5 non-overlapping batches of the size of 7.000, which is around equal to the training dataset. For each batch we evaluated the percentage of peptides predicted as positive (Pred-P) or negative (Pred-N) and the Wasserstein distance (WD) for the descriptors Charge at pH 7, hydrophobic moment and hydrophobicity.

Table 9: Stability analysis for the sequences generated by the WAE.

Batch	Pred-P	Pred-N	WD-charge	WD-hm	WD-hp
**1**	66.3	2.36	0.416	0.015	0.038
**2**	67.14	2.16	0.455	0.012	0.036
**3**	65.83	2.4	0.412	0.013	0.041
**4**	66.27	2.43	0.44	0.013	0.034
**5**	67.19	2.39	0.438	0.014	0.042

Additional t-SNE visualizations

The two figures below show that the RNN is overfitting as intended, because no regularization was applied. The sequence space generated by the RNN is less densely populated than that of the VAE-LIN model. This observation indicates that RNN-generated sequences closely resemble, or are identical to, the training data. In contrast, these results suggest that the autoencoder does not exhibit overfitting.

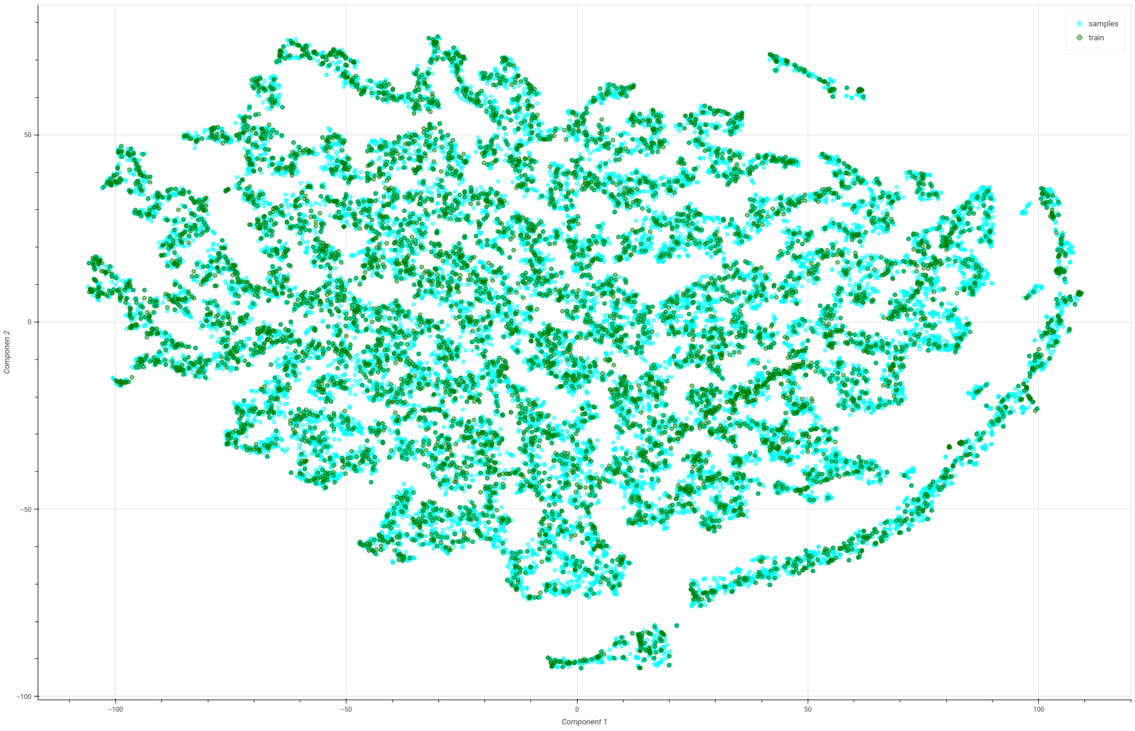

Figure 12: Visualization for RNN-generated sequences.

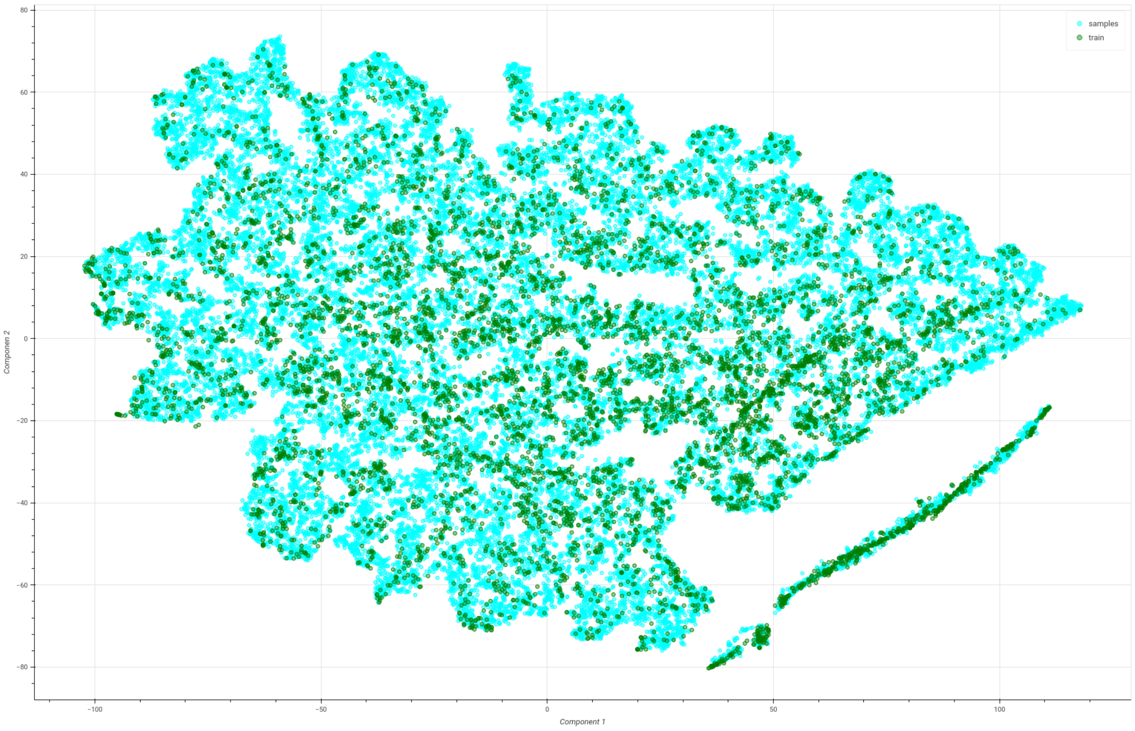

Figure 13: Visualization for sequences generated by the VAE-LIN model.
